# Nine New Farnesylphenols from the Basidiomycete *Albatrellus Caeruleoporus*

**DOI:** 10.1007/s13659-014-0015-5

**Published:** 2014-04-23

**Authors:** Liang-Yan Liu, Zheng-Hui Li, Gang-Qiang Wang, Kun Wei, Ze-Jun Dong, Tao Feng, Gen-Tao Li, Yan Li, Ji-Kai Liu

**Affiliations:** 1 State Key Laboratory of Phytochemistry and Plant Resources in West China, Kunming Institute of Botany, Chinese Academy of Sciences, Kunming, 650201 Yunnan China; 2 University of the Chinese Academy of Sciences, Beijing, 100049 China

**Keywords:** *Albatrellus caeruleoporus*, Mushroom, Polyporaceae, Farnesylphenols

## Abstract

Nine previously-unreported farnesylphenols, involving eight neogrifolin derivatives (**1**–**8**) and one grifolin analogue (**9**), together with three known compounds, were isolated from the fruiting bodies of the mushroom *Albatrellus caeruleoporus*. Their structures were elucidated as (*S*)-17-hydroxy-18,20-ene-neogrifolin (**1**), (*S*)-18,19-dihydroxyneogrifolin (**2**), (*S*)-9-hydroxy-10,22-ene-neogrifolin (**3**), (9*S*,10*R*)-6,10-epoxy-9-hydroxyneo grifolin (**4**), (9*S*,10*R*)-6,9-epoxy-10-hydroxyneogrifolin (**5**), (−)-13,14-dihydroxyneogrifolin (**6**), albatrelin G (**7**), albatrelin H (**8**), and one grifolin analogue, (*S*)-10-hydroxygrifolin (**9**), grifolin (**10**), neogrifolin (**11**), and albatrellin (**12**) by extensive spectroscopic analyses and chemical methods. Compounds **7** and **8** showed weak cytotoxic activity to cell lines HL-60, SMMC-7721, A-549, and MCF-7, in vitro.

**Electronic supplementary material:**

The online version of this article (doi:10.1007/s13659-014-0015-5) contains supplementary material, which is available to authorized users.

## Introduction

Mushrooms of the *Albatrellus* genus are well known for producing farnesylphenols, such as grifolin, neogrifolin and their derivatives [1–6]. Farnesylphenols can be divided into two groups: monomers of grifolin and neogrifolin derivatives, and dimers of them. The monomers were reported to possess diverse biological activities, such as anti-oxidative activity [3], anti-microbial effect [7, 8], promotion of melanin synthesis [9], activity on human and rat vanilloid receptor 1 [10] inhibition of tumor-cell growth [11], and inhibition of nitric oxide production in RAW 264.7 cells [4]. And the dimers (fungal pigments) are regarded as the chemical base of the conspicuous fruiting bodies of these mushrooms [2, 4].

*Albatrellus caeruleoporus* is a nontoxic and inedible mushroom distributed in central and southwestern China. Its fruiting body is white with a light blue skin on the pileus [12]. Previous investigation on *A. caeruleoporus* led to three grifolin monomers, grifolin, neogrifolin, and grifolinone A, and one dimer, grifolinone B [4]. Their nitorite production inhibitory activities were reported [4]. In order to find more farnesylphenols with biological activities a systematic phytochemical investigation on the basidiomycete *A. caeruleoporus* was performed, it led to isolate eight new neogrifolin derivatives (**1**–**8**), a new grifolin analogue (**9**), grifolin (**10**) [10], neogrifolin (**11**) [10], and albatrellin (**12**) [2]. Their structures were identified by a combination of extensive spectroscopic analyses (NMR, MS, IR, UV, and [α]_D_) and chemical methods. Compounds **1**–**9** were oxygenated farnesylphenols, which have not previously been reported in the *Albatrellus* genus, and might be regarded as a chemotaxonomic evidence for identification of this mushroom. All new compounds were tested in a cytotoxicity assay in vitro against five human cancer cell lines.

## Results and Discussion

**Fig. 1:**
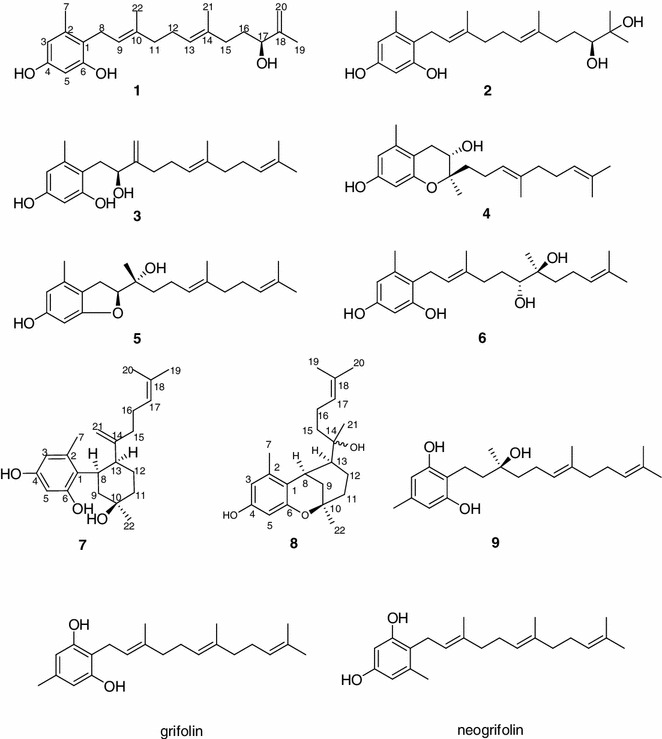
Chemical structures of compounds **1**–**9**

The chloroform–methanol (1:1) extract of fruiting bodies of *A. caeruleoporus* was subjected to silica gel, RP-18, Sephadex LH-20 column chromatography (CC), and semipreparative HPLC purification steps to give compounds **1**–**12** (Fig. 1). Compounds **1**–**8**, namely (*S*)-17-hydroxy-18,20-ene-neogrifolin (**1**), (*S*)-18,19-dihydroxyneogrifolin (**2**), (*S*)-9-hydroxy-10,22-ene-neogrifolin (**3**), (9*S*,10*R*)-6,10-epoxy-9-hydroxyneogrifolin (**4**), (9*S*,10*R*)-6,9-epoxy-10-hydroxyneogrifolin (**5**), (−)-13,14-dihydroxyneogrifolin (**6**), albatrelin G (**7**), albatrelin H (**8**), were neogrifolin derivatives, (*S*)-10-hydroxygrifolin (**9)** was a grifolin analogue, and compound **12** was a violet pigment named albatrellin.

**Fig. 2:**
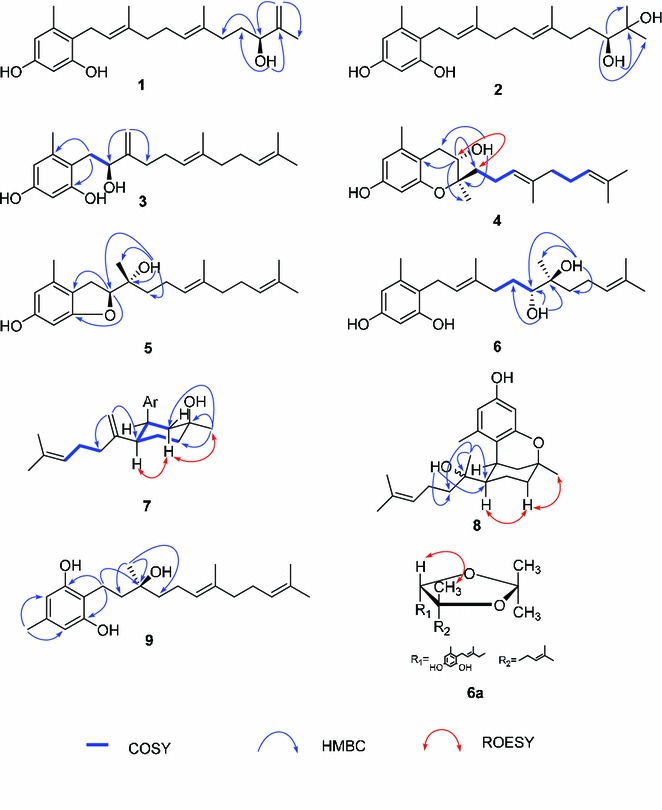
Selective 2D NMR correlations for compounds **1**–**9**

Compound **1**, colorless oil, displayed a [M]^+^ ion peak at *m/z* 344.2348 in positive HREIMS, corresponding to the molecular formula C_22_H_32_O_3_ and seven degrees of unsaturation. The IR spectrum showed absorption at 3421 cm^−1^ which indicated the presence of OH groups. The ^1^H NMR spectrum contained signals for two *m*-coupling aromatic protons at *δ*_H_ 6.25 (1H, d, *J* = 1.8 Hz) and 6.17 (1H, d, *J* = 1.8 Hz), four olefinic protons, and four singlet methyls. Combined with ^13^C NMR (DEPT) experiment, the existence of a 1,2,4,6-tetra-substituted phenyl ring, a terminal double bond, two tri-substituted double bonds, one oxygen-bearing methine, five methylenes, and four methyls were assigned. The ^1^H and ^13^C NMR spectroscopic data were close to those of neogrifolin, except that a methylene and a methyl group in neogrifolin were replaced by a hydroxyl methine (*δ*_C_ 75.3) and a terminal double bond (*δ*_C_ 110.3 and 149.3), respectively. According to the observed HMBC correlations from *δ*_H_ 6.25 and 6.17 (H-20) to *δ*_C_ 17.8 (C-19) and 75.3 (C-17), the terminal double bond was located at the end of the farnesyl side chain, and the hydroxyl group was at C-17 (*δ*_C_ 75.3). This conclusion was supported by cross peaks from *δ*_H_ 3.73 (OH-17) to *δ*_C_ 34.5 (C-16), 75.3 (C-17) and 149.3 (C-18), and from 3.97 (H-17) to *δ*_C_ 36.4 (C-15) in HMBC spectrum (Fig. 2). The absolute configuration of the only chiral center (C-17) of **1** was deduced to be *S* by comparing the optical rotation value of **1** ([α]_D_^21^ −9.0, MeOH) with that of (*R*)-(+)-3-methyl-3-buten-2-ol ([α]_D_ +7.6, CHCl_3_) [13]. Therefore, compound **1** was elucidated and named as (*S*)-17-hydroxy-18,20-ene-neogrifolin.

Compound **2** possessed a molecular formula of C_22_H_34_O_4_ according to HREIMS which showed a molecular ion peak at *m/z* 362.2452, requiring six degrees of unsaturation. Inspection of the ^1^H and ^13^C NMR (DEPT) spectra indicated five methyls, five methylenes, four *sp*^2^ methines, one oxygen-bearing methine, and seven quaternary carbons. The 1D NMR spectroscopic data were similar to those of **1**, except for the terminal double bond being replaced by a methyl and an oxygen-bearing quaternary carbon, which was confirmed by HMBC correlations from *δ*_H_ 3.54 (OH-17) to *δ*_C_ 72.9 (C-18), from *δ*_H_ 3.24 (H-17) to *δ*_C_ 25.8 (C-19 and C-20), from *δ*_H_ 1.11 (H-20) to *δ*_C_ 25.8 (C-19), 72.9 (C-18) and 78.5 (C-17). The absolute configuration of C-17 in **2** was assigned to be *S*, the same as **1**, on a biogenetic point of view. And this supposition was further confirmed by a comparison of the optical rotation values between **2** ([α]_D_^21^ −9.2, MeOH) and (*R*)-2-methylpentane-2,3-diol ([α]_D_^18.5^ +27.3, ether) [14]. Therefore, compound **2** was identified as (*S*)-18,19-dihydroxyneogrifolin.

Compound **3** was determined to have the molecular formula of C_22_H_32_O_3_ from HREIMS at *m/z* 344.2364 ([M]^+^). The ^13^C NMR (DEPT) spectra showed signals of a tetra-substituted phenyl moiety, a terminal double bond, two tri-substituted double bonds, a hydroxyl methine, four methyls, and five methylenes, which resembled those of compound **1**. Extensive 2D NMR (COSY, ROESY and HMBC) analyses revealed that the locations of the double bond and the oxygen-bearing methine were different with those of **1**. COSY correlations from *δ*_H_ 2.82 and 2.68 (H-8) to *δ*_H_ 4.28 (*δ*_C_ = 77.0) suggested that C-9 was the oxygenated carbon. Moreover, *δ*_H_ 5.11 and 4.84 (*δ*_C_ = 108.9, t) gave HMBC correlations to *δ*_C_ 32.5 (C-11) and 77.0 (C-9), revealing that the Me-22 in compound **1** converted to be a double bond in **3**. Therefore, the structure of compound **3** was identified as 9-hydroxy-10,22-ene-neogrifolin. The absolute stereochemistry of the chiral center of C-9 was determined to be *S* by comparing the optical rotation value of **3** ([α]_D_ −8.8, MeOH) with (*S*)-3-methyl-1-phenylbutan-2-ol ([α]_D_ −29.5, CHCl_3_) [15].

The HREIMS of compound **4** showed a [M]^+^ ion peak at *m/z* 344.2348, indicating a molecular formula of C_22_H_32_O_3_ and seven degrees of unsaturation. A comparison of the MS and 1D NMR data of **4** with those of **3** revealed that **4** was another neogrifolin analogue resembled **3** except for the double bond between C-10 and C-22 in **3** being replaced by a methyl (Me-22) and an oxygenated quaternary carbon (C-10) in **4**. This structure requires six degrees of unsaturation, and an additional ring was needed to complete the unsaturation. There were two plausible proposals: an epoxy ring between C-6 and C-9 or between C-6 and C-10. HMBC correlations from H-9 (*δ*_H_ 3.85) to C-1 (*δ*_C_ 111.0), C-10 (*δ*_C_ 78.6), C-11 (*δ*_C_ 38.7), and C-22 (*δ*_C_ 17.9), and from the OH at *δ*_H_ 4.14 to C-8 (*δ*_C_ 29.7), C-9 (*δ*_C_ 68.4) and C-10 (*δ*_C_ 78.6) revealed the location of the free OH at C-9, placing the epoxy ring between C-6 and C-10. Therefore, the planar structure of **4** was elucidated as 6,10-epoxy-9-hydroxyneogrifolin. The ROESY spectrum displayed cross peaks of H-9/H-11 and H-9/H-12, suggesting the same orientation of H-9 and the geranyl group. From a biogenetic point of view, compounds **4** and **3** should share the same absolute configuration on C-9. So the absolute stereochemistry of compound **4** was deduced to be 9*S*, 10*R*.

Compound **5** was proposed to be a neogrifolin derivative on basis of HREIMS which displayed the molecular ion peak at *m/z* 344.2358. A comparison of the ^13^C NMR (DEPT) spectra of **5** with those of **4** revealed the resemblance of the two structures, for example, the presence of the 1-(2-methyl-4,6-dihydroxyl)-phenyl group and the geranyl moiety. The structural difference between compounds **4** and **5** was the fragment from C-8 to C-10, according to the different chemical shifts of the corresponding carbons and protons (Tables 1 and 2). In order to establish the structure of **5**, extensive 2D NMR experiments were employed. The HMBC correlations of H-9/C-1 and H-9/C-6 indicated the existence of an oxygen bridge between C-6 and C-9. The free hydroxyl group was determined to be located at C-10 by HMBC correlations from OH-10 to C-9, C-10, C-11 and C-22. Biogenetically speaking, compound **5** would show the same stereochemistry at C-9 and C-10 as compound **4**. Therefore, compound **5** was elucidated as (9*S*,10*R*)-6,9-epoxy-10-hydroxyneogrifolin.

**Table 1:** ^1^H NMR spectroscopic data for compounds **1**–**6** in acetone-*d*_6_ (*δ* in ppm, *J* in Hz)

No	**1** ^a^	**2** ^c^	**3** ^a^	**4** ^b^	**5** ^a^	**6** ^c^
**3**	6.17, d (1.8)	6.17, d (2.3)	6.21, s	6.25, d (2.1)	6.12, br. s	6.17, s
**5**	6.25, d (1.8)	6.25, d (2.3)	6.22, s	6.12, d (2.1)	6.03, br. s	6.25, s
**7**	2.15, s	2.15, s	2.20, s	2.11, s	2.11, s	2.15, s
**8**	3.26, d (6.7)	3.26, d (6.7)	2.82, dd (14.4, 2.2)	2.79, dd (16.1, 5.9)	3.06, dd (15.3, 7.9)	3.27*
			2.68, dd (14.4, 9.3)	2.45, dd (16.1, 8.5)	2.93, dd (15.3, 9.5)	
**9**	5.09, t (6.7)	5.09, t (6.7)	4.28, dd (9.3, 2.2)	3.85, td (8.5, 5.8)	4.62, dd (9.5, 7.9)	5.11*
**11**	1.90–2.00, m	1.97–1.99, m	2.22–2.25*	1.66–1.75, m	1.50–1.56, m	2.25, t (9.9)
			2.14–2.17, m			
						1.96–2.03*
**12**	2.03–2.11, m	2.07–2.10, m	2.22–2.25*	2.14–2.24, m	2.09–2.20, m	1.69–1.74, m
						1.33–1.40, m
**13**	5.12, t (6.6)	5.14, t (7.0)	5.20, br. s	5.17, t (7.2)	5.18, t (6.9)	3.29*
**15**	1.90–2.00, m	2.21–2.26, m	1.98, t (7.5)	1.97, t (7.6)	1.98, t (7.5)	1.54–1.58, m
		1.94–1.97, m				1.33–1.40, m
**16**	1.54–1.58, m	1.62–1.68, m	2.07–2.09*	2.06–2.08*	2.06–2.09*	2.09–2.13, m
		1.28–1.35, m				2.03–2.05, m
**17**	3.97, m	3.24, td (5.3, 1.8)	5.10*	5.10, t (6.9)	5.10, t (7.0)	5.11*1
**19**	1.68, s	1.11, s	1.59, s	1.58, s	1.59, s	1.58, s
**20**	4.89, br. s	1.11, s	1.65, s	1.65, s	1.65, s	1.64, s
	4.74, br. s					
**21**	1.58, s	1.58, s	1.63, s	1.61, s	1.63, s	1.07, s
**22**	1.75, s	1.75, s	5.11, br. s	1.16, s	1.20, s	1.76, s
			4.84, br. s			
**4-OH**	7.86, s	7.88, s	8.01, s	7.89, s	8.05, s	7.89, s
**6-OH**	8.02, s	8.06, s	8.56, s			8.05, s
**9-OH**			4.88, d (2.8)	4.14, d (5.6)		
**10-OH**					3.54, s	
**13-OH**						3.56, d (5.8)
**14-OH**						3.20, s
**17-OH**	3.73, d (4.2)	3.54, d (5.3)				
**18-OH**		3.40, s				

^a^Measured at 400 MHz

^b^Measured at 500 MHz

^c^Measured at 600 MHz

* Signals were overlapped

**Table 2:** ^13^C NMR spectroscopic data for compounds **1**–**6** in acetone-*d*_6_ (*δ* in ppm)

No	**1** ^a^	**2** ^c^	**3** ^a^	**4** ^b^	**5** ^a^	**6** ^c^
**1**	118.3, C	118.3, C	117.1, C	111.0, C	117.8, C	118.3, C
**2**	138.9, C	138.9, C	139.0, C	138.6, C	135.2, C	138.8, C
**3**	109.4, CH	109.3, CH	109.8, CH	110.1, CH	108.5, CH	109.3, CH
**4**	156.5, C	156.5, C	157.1, C	157.1, C	158.4, C	156.5, C
**5**	101.0, CH	101.0, CH	102.2, CH	102.0, CH	95.2, CH	101.0, CH
**6**	156.4, C	156.4, C	157.9, C	154.7, C	161.6, C	156.4, C
**7**	19.9, CH_3_	19.9, CH_3_	20.5, CH_3_	19.3, CH_3_	19.0, CH_3_	19.9, CH_3_
**8**	25.1, CH_2_	25.1, CH_2_	34.4, CH_2_	29.7, CH_2_	29.1, CH_2_	25.1, CH_2_
**9**	124.8, CH	124.8, CH	77.0, CH	68.4, CH	89.8, CH	124.4, CH
**10**	134.1, C	134.1, C	153.4, C	78.6, C	73.3, C	134.6, C
**11**	40.4, CH_2_	40.4, CH_2_	32.5, CH_2_	38.7, CH_2_	39.3, CH_2_	37.8, CH_2_
**12**	27.2, CH_2_	27.2, CH_2_	27.3, CH_2_	22.1, CH_2_	22.5, CH_2_	30.2, CH_2_
**13**	124.8, CH	124.8, CH	125.1, CH	125.4, CH	125.6, CH	77.8, CH
**14**	135.5, C	135.8, C	135.7, C	135.4, C	135.3, C	74.2, C
**15**	36.4, CH_2_	37.6, CH_2_	40.4, CH_2_	40.4, CH_2_	40.4, CH_2_	38.5, CH_2_
**16**	34.5, CH_2_	30.7, CH_2_	27.3, CH_2_	27.4, CH_2_	27.3, CH_2_	22.6, CH_2_
**17**	75.3, CH	78.5, CH	125.1, CH	125.1, CH	125.1, CH	126.2, CH
**18**	149.3, C	72.9, C	131.6, C	131.6, C	131.6, C	131.1, C
**19**	17.8, CH_3_	25.8, CH_3_	17.7, CH_3_	17.7, CH_3_	17.7, CH_3_	17.6, CH_3_
**20**	110.3, CH_2_	25.8, CH_3_	25.8, CH_3_	25.8, CH_3_	25.8, CH_3_	25.8, CH_3_
**21**	16.1, CH_3_	16.1, CH_3_	16.1, CH_3_	16.0, CH_3_	16.0, CH_3_	22.8, CH_3_
**22**	16.1, CH_3_	16.1, CH_3_	108.9, CH_2_	17.9, CH_3_	22.3, CH_3_	16.3, CH_3_

^a^Measured at 100 MHz

^b^Measured at 125 MHz

^c^Measured at 150 MHz

Compound **6** was obtained as a colorless oil, with a molecular formula of C_22_H_34_O_4_ according to the HREIMS at *m/z* 362.2432 ([M]^+^). Inspection of the ^1^H, ^13^C (DEPT) and HSQC NMR spectra allowed the assignment of five methyls, five methylenes, five methines, seven quaternary carbons, and four active protons. Comparing the ^1^H and ^13^C NMR spectroscopic data of **6** with those of neogrifolin indicated that compound **6** shared the “1-(2-methyl-4,6-dihydroxyl)-phenyl” partial structure with neogrifolin, but had a different side chain, in which one double bond in the farnesyl group was replaced by two oxygen-bearing *sp*^3^ carbons. The COSY cross peaks of H-11/H-12/H-13, and HMBC correlations from OH at *δ*_H_ 3.56 to C-12, C-13 and C-14, and from *δ*_H_ 3.20 to C-21, C-13, C-14, and C-15 (Fig. 2) suggested the oxygenated carbons being located at C-13 (*δ*_C_ 77.3, CH) and C-14 (*δ*_C_ 74.2, C). In order to identify the relative configuration of the two chiral centers C-13 and C-14, compound **6** was reacted with 2,2-dimethoxypropane in DMF for 30 min at room temperature to yield its di-*O*-isopropylidene derivative **6a**. The observed ROESY correlations of Me-21/H-13 (Fig. 2) indicated that the 13,14-diol existed as the *erythro* form. So, the absolute configuration of **6** should be 13*S*, 14*S* or 13*R*, 14*R*.

Compound **7** possessed a molecular formula of C_22_H_32_O_3_ from its HREIMS, which displayed a molecular ion peak at *m/z* 344.2348. A comparison of the ^1^H and ^13^C NMR data of **7** with those of **1** revealed the presence of the 1-(2-methyl-4,6-dihydroxyl)-phenyl group. Combined with MS spectral data, compound **7** was determined to be a neogrifolin derivative unambiguously. Unlike the other neogrifolin analogues (**1**–**6)** which had straight-chains as “tails”, compound **7** had a cyclohexane moiety—by C-8 connecting to C-13—in its tail. It was supported by COSY correlations of H-9/H-8/H-13/H-12/H-11, and HMBC cross peaks from Me-22 to C-9, C-10 and C-11 (Fig. 2). The remaining part of the “tail” was a 2-(6-methyl)-1,5-heptadiene residue. This residue was connected with C-13, because COSY correlations of H-15/H-16/H-17, and HMBC correlations from H-21 to C-13 and C-15, and from Me-19 and -20 to C-18 and C-17 (Fig. 2) were observed. In order to determine the relative stereochemistry of C-8, C-10 and C-13, a ROESY experiment was performed, combined with further analysis of the coupling constants of several signals in ^1^H NMR spectrum. The observed broad singlet (br. s) signal of H-8 (*δ*_H_ = 3.22) in its ^1^H NMR spectrum indicated that H-8 existed as an equatorial bond in the stable boat conformation of the cyclohexane moiety, as shown in Fig. 2. Likewise, H-13 was proposed to be in an axial position because of the doublet of triplets at *δ*_H_ 3.22 with coupling constants of 12.5 and 2.7 Hz, respectively. Furthermore, The ROESY correlations of H-13/H_ax_-9 and Me-22/H_ax_-9 revealed the same orientation of H-13 and Me-22. Therefore, H-8, H-13 and Me-22 were deduced to be *α*-, *α*-, *α*- orientated. In compound **7**, a ring was formed by new C–C bond connection between C-8 and C-13 in side chain.

Compound **8** exhibited a molecular ion peak at *m/z* 344.2345 in HREIMS, indicating the molecular formula of C_22_H_32_O_3_ which required seven degrees of unsaturation. According to the ^1^H and ^13^C NMR (DEPT) spectra, 22 carbon signals were recognized as five methyls, four methylenes, five methines, and seven quaternary carbons. Extensive NMR analyses suggested that the structure of **8** resembled that of **7**, except for the terminal double bond C-14=C-21 in **7** being saturated to be a methyl and an oxygen-bearing quaternary carbon, which was confirmed by HMBC correlations from *δ*_H_ 1.13 (Me-21) to *δ*_C_ 36.8 (C-15), 55.9 (C-13), and 74.0 (C-14), and from *δ*_H_ 3.30 (OH-14) to *δ*_C_ 36.8 (C-15), 55.9 (C-13), and 74.0 (C-14). So far, six degrees of unsaturation was assigned, and one more ring should be constructed to complete the structure of **8**. The only possible ring to be formed was the oxygen bridge between C-6 and C-10. The stereochemistry of C-13 was identified by analysis of ^1^H NMR spectrum, in which H-13 showed a doublet-of-triplets peak with the coupling constants of 12.8 and 2.0 Hz, respectively, suggesting the axial bond of H-13. Me-22 had the same orientation as H-13 by the observed ROESY correlations of H-13/H_ax_-11/Me-22, and because of the planar structure of the phenyl group, H-8 and Me-22 should be on the same orientation. Thus, H-8, H-13 and Me-22 were determined to be *α*-, *α*-, *α*- orientated, the same as for compound **7**.

Compound **9** was proposed to possess a molecular formula of C_22_H_34_O_3_ on basis of HREIMS at *m/z* 346.2505 ([M]^+^). Its ^13^C NMR (DEPT) spectrum showed 20 carbon signals, including two signals at *δ*_C_ 108.4 (CH) and 156.7 (C) which represented two carbons respectively. The overlapped carbon signals indicated that **9** was a grifolin derivative possessing a symmetric aromatic ring, which was confirmed by HMBC cross peaks from *δ*_H_ 2.10 (Me-7) to *δ*_C_ 108.4 (C-3 and -5) and 136.5 (C-4), from *δ*_H_ 2.67 (H-8) to *δ*_C_ 113.9 (C-1) and 156.7 (C-2 and -6), and from *δ*_H_ 8.05 (OH-2 and -6) to *δ*_C_ 108.4 (C-3 and -5), 113.9 (C-1) and 156.7 (C-2 and -6). Besides the aromatic ring, the remaining signals represented an oxygenated farnesyl group with four methyls, six methylenes, two pairs of tri-substituted double bonds, and one oxygenated quaternary carbon. The next problem to be resolved was the position of oxygenation, which was addressed by 2D NMR (HMBC and COSY) experiments. The HMBC correlations from *δ*_H_ 2.67 (H-8) to *δ*_C_ 72.6 (C-10), and from *δ*_H_ 1.20 (Me-22) to *δ*_C_ 41.2 (C-9), 72.6 (C-10) and 42.6 (C-11) revealed the hydroxylation of C-10. Hence, the planar structure of **9** was established as 10-hydroxygrifolin. The absolute stereochemistry of C-10 was deduced to be *S* by a comparison of the optical rotation value of **9** ([*α*]_D_ −8.7, MeOH) with that of (*S*)-3-methyl-1-phenyl-3-pentanol ([*α*]_D_ −1.6, CHCl_3_) [16].

All the new compounds were assayed for their cytotoxicity against five human cancer cell lines (HL-60, SMMC-7712, A-549, MCF-7, and SW480) by the MTT method in vitro, with DDP and taxol as positive controls. Compound **7** showed cytotoxic activities to cell lines HL-60, SMMC-7721, A-549, and MCF-7, with IC_50_ of 12.8, 33.8, 33.0, and 33.2 *μ*M, respectively, and **8** exhibited weak growth inhibition activity to human tumor cell lines HL-60 and A-549, with IC_50_ of 21.8 and 30.3 *μ*M, respectively.

## Experimental Section

### General Experimental Procedures

Optical rotations were measured on a Jasco model 1020 polarimeter (Jasco International Co. Ltd, Tokyo, Japan). UV spectra were recorded on a Shimadzu double-beam 2401A spectrophotometer (Shimadzu, Kyoto, Japan). IR spectra were obtained on a Bruker Tensor 27 FT-IR spectrometer (Bruker, Ettlingen, Germany) using KBr pellets. 1D and 2D NMR spectra were acquired on Bruker AV-600, DRX-500 and AM-400 instruments at room temperature with TMS as internal standard (Bruker, Rheinstetten, Germany). Chemical shifts (*δ*) were expressed in ppm with reference to the solvent signals. Mass spectra (MS) were recorded on a VG Autospec-3000 spectrometer (VG, Manchester, England). Silica gel (200–300 mesh, Qingdao Marine Chemical Inc., Qingdao, China), Sephadex LH-20 (Amersham Biosciences, Sweden), and RP-18 gel (40–75 μm, Fuji Silysia Chemical Ltd. Japan) were used for CC. HPLC analysis (Zorbax SB-C18, 5 μm, 4.6 × 150 mm) was performed on an Agilent 1100 liquid chromatography system, and semi-preparative HPLC was performed on an Agilent 1200 liquid chromatography system equipped with a Zorbax SB-C_18_ column (9.4 mm × 150 mm). Pre-coated silica gel GF254 plates (Qingdao Marine Chemical Inc., Qingdao, China) were used for TLC. Fractions were monitored by TLC, and spots were visualized by heating silica gel plates sprayed with 10 % H_2_SO_4_ in ethanol.

### Fungal Material

The fungus *A. caeruleoporus* was collected in Anhui province, China, in October, 2011. The voucher specimen (GDGM 29146) has been deposited in the Herbarium of Microbiology Institute of Guangdong.

### Extraction and Isolation

The dried fruiting bodies of *A. caeruleoporus* (about 200 g) were extracted with chloroform/methanol (1/1) for three times (5 L × 3). Evaporation of the solvent under reduced pressure gave the crude extract (20 g), which was subjected to silica gel CC using a petroleum ether–acetone gradient (1:0 → 0:1) to afford fractions A–E. Fraction B was purified by CC over silica gel with a petroleum ether–acetone system (20:1 → 10:1) to yield two fractions B_1_ and B_2_. Fraction B_1_ was purified by semi-preparative HPLC (CH_3_CN/H_2_O, 6:4) to gave **1** (5.2 mg) and **10** (12.0 mg), while B_2_ was applied on a Sephadex LH-20 (CHCl_3_/MeOH 1/1) column and then on semi-preparative HPLC eluting with MeCN-H_2_O (6:4) to yield compounds **2** (6.0 mg) and **11** (21.0 mg). Fraction C was subjected to CC with RP-18 silica gel eluting with 90 % methanol, and then purified by semi-preparative HPLC (CH_3_CN/H_2_O, 55:45) to get compound **6** (5.1 mg). Fraction D was submitted to silica gel CC eluting with petroleum ether-acetone gradient (15:1) to gave two fractions D_1_ and D_2_, which were purified first by PR-18 and then by Sephadex LH-20 CC to yield fractions D_1_′ and D_2_′, respectively. Fraction D_1_′ was loaded on a semi-preparative HPLC eluting with MeCN-H_2_O (60:40 → 65:35) to afford compounds **9** (4.8 mg) and **3** (4.5 mg), while fraction D_2_′ was passed through a semi-preparative HPLC (MeCN/H_2_O, 60:40 → 65:35) to yield compounds **8** (6.4 mg) and **5** (4.0 mg). Fraction E was applied on CC over RP-18 to give fractions E_1_ and E_2_. Compound **4** (6.5 mg) was obtained from fraction E_1_ which was passed through silica gel column (petroleum ether-acetone, 10:1), Sephadex LH-20 column (chloroform–methanol, 1:1), and semi-preparative HPLC (MeCN/H_2_O 7:3), successively. Fraction E_2_ was passed through Sephadex LH-20, and then loaded on semi-preparative HPLC to yield compound **7** (4.5 mg).

### (*S*)-17-hydroxy-18,20-ene-neogrifolin (**1**)

Colorless oil; [α]_D_^21^ −9.0 (*c* 0.20, MeOH); UV *λ*_max_ (MeOH) (log *ε*) 283 (2.67) nm; IR (KBr) *ν*_max_ 3421, 3075, 2970, 2922, 2855, 1611, 1447, 1140 cm^−1^; ^1^H and ^13^C NMR spectroscopic data, see Tables 1 and 2; EI-MS *m/z*: 344 [M]^+^, 326 [M–H_2_O]^+^, 191, 175, 137; HR-EI-MS *m/z*: 344.2348 [M]^+^ (calcd for C_22_H_32_O_3_, 344.2351).

### (*S*)-18,19-dihydroxyneogrifolin (**2**)

Colorless oil; [α]_D_^21^ −9.2 (*c* 0.18, MeOH); UV *λ*_max_ (MeOH) (log *ε*) 282 (2.67) nm; IR (KBr) *ν*_max_ 3423, 2974, 2925, 2855, 1613, 1467, 1141 cm^−1^; ^1^H and ^13^C NMR spectroscopic data, see Tables 1 and 2; EI-MS *m/z*: 363 [M+H]^+^, 362 [M]^+^, 344 [M–H_2_O]^+^, 191, 175, 137; HR-EI-MS *m/z*: 362.2452 [M]^+^ (calcd for C_22_H_34_O_4_, 362.2457).

### (*S*)-9-hydroxy-10,22-ene-neogrifolin (**3**)

Colorless oil; [α]_D_^21^ −8.8 (*c* 0.24, MeOH); UV *λ*_max_ (MeOH) (log *ε*) 283 (2.90) nm; IR (KBr) *ν*_max_ 3420, 2966, 2923, 2855, 1614, 1447, 1143 cm^−1^; ^1^H and ^13^C NMR spectroscopic data, see Tables 1 and 2; EI-MS *m/z*: 344 [M]^+^, 326 [M–H_2_O]^+^, 137; HR-EI-MS *m/z*: 344.2364 [M]^+^ (calcd for C_22_H_32_O_3_, 344.2351).

### (9*S*,10*R*)-6,10-epoxy-9-hydroxyneogrifolin (**4**)

Colorless oil; [α]_D_^22^ −6.7 (*c* 0.28, MeOH); UV *λ*_max_ (MeOH) (log *ε*) 282 (2.74) nm; IR (KBr) *ν*_max_ 3422, 3038, 2967, 2923, 2854, 1618, 1601, 1460, 1138 cm^−1^; ^1^H and ^13^C NMR spectroscopic data, see Tables 1 and 2; EI-MS *m/z*: 344 [M]^+^, 191, 137; HR-EI-MS *m/z*: 344.2348 [M]^+^ (calcd for C_22_H_32_O_3_, 344.2351).

### (9*S*,10*R*)-6,9-epoxy-10-hydroxyneogrifolin (**5**)

Colorless oil; [α]_D_^22^ −8.6 (*c* 0.19, MeOH); UV *λ*_max_ (MeOH) (log *ε*) 282 (2.82) nm; IR (KBr) *ν*_max_ 3513, 3405, 2969, 2922, 2856, 1629, 1602, 1495, 1449, 1128 cm^−1^; ^1^H and ^13^C NMR spectroscopic data, see Tables 1 and 2; EI-MS *m/z*: 344 [M]^+^, 191, 175, 150; HR-EI-MS *m/z*: 344.2358 [M]^+^ (calcd for C_22_H_32_O_3_, 344.2351).

### (−)-13,14-Dihydroxyneogrifolin (**6**)

Colorless oil; [α]_D_^22^ −9.2 (*c* 0.20, MeOH); UV *λ*_max_ (MeOH) (log *ε*) 282 (2.65) nm; IR (KBr) *ν*_max_ 3440, 2969, 2924, 2856, 1628, 1452, 1141 cm^−1^; ^1^H and ^13^C NMR spectroscopic data, see Tables 1 and 2; EI-MS *m/z*: 362 [M]^+^, 344 [M–H_2_O]^+^, 326 [M–2 × H_2_O]^+^, 191, 175, 137; HR-EI-MS *m/z*: 362.2432 [M]^+^ (calcd for C_22_H_34_O_4_, 362.2457).

### Albatrelin G (**7**)

Colorless oil; [α]_D_^22^ −15.2 (*c* 0.19, MeOH); UV *λ*_max_ (MeOH) (log *ε*) 283 (2.93) nm; IR (KBr) *ν*_max_ 3441, 2969, 2929, 2855, 1640, 1615, 1495, 1452, 1141 cm^−1^; ^1^H and ^13^C NMR spectroscopic data, see Table 3; EI-MS *m/z*: 344 [M]^+^, 326 [M–H_2_O]^+^, 175; HR-EI-MS *m/z*: 344.2348 [M]^+^ (calcd for C_22_H_32_O_3_, 344.2351).

**Table 3:** ^1^H and ^13^C NMR spectroscopic data for compounds **7**–**9** in acetone-*d*_6_ (*δ* in ppm, *J* in Hz)

No	**7** ^b^		**8** ^a^		**9** ^a^	
	*δ*_C_, type	*δ*_H_ (*J* in Hz)	*δ*_C_, type	*δ*_H_ (*J* in Hz)	*δ*_C_, type	*δ*_H_ (*J* in Hz)
**1**	113.7, C		115.6, C		113.9, C	
**2**	139.1, C		139.1, C		156.7, C	
**3**	109.3, CH	6.11, d (2.3)	109.6, CH	6.13, d (2.2)	108.4, CH	6.19, s
**4**	157.0, C		157.0, C		136.5, C	
**5**	100.6, CH	6.09, d (2.3)	100.7, CH	6.07, d (2.2)	108.4, CH	6.19, s
**6**	158.6, C		158.6, C		156.7, C	
**7**	20.0, CH_3_	2.01, s	20.9, CH_3_	2.36, s	21.2, CH_3_	2.10, s
**8**	33.9, CH	3.22, br. s	30.3, CH	3.52, br. s	18.2, CH_2_	2.67, m
**9**	38.9, CH_2_	1.97–2.00, dd (12.8, 3.0)	40.4, CH_2_	1.87, dd (12.8, 3.2)	41.2, CH_2_	1.66–1.70, m
		1.69–1.73, dd (12.8, 3.0)		1.62, dd (12.8, 8.0)		
**10**	74.2, C		74.5, C		72.6, C	
**11**	40.8, CH_2_	1.89, br. d (11.0)	41.5, CH_2_	1.89–1.92, m	42.6, CH_2_	1.50–1.54, m
		1.62–1.64, m		1.53–1.56*		
**12**	24.6, CH_2_	1.49–1.55, m	21.6, CH_2_	1.53–1.56*	23.3, CH_2_	2.10–2.15, m
		1.20–1.34, m		1.31–1.35, m		
**13**	48.7, CH	2.34, dt (12.5, 2.7)	55.9, CH	1.76, dt (12.8, 2.0)	125.9, CH	5.15, t (6.9)
**14**	152.5, C		74.0, C		135.0, C	
**15**	37.4, CH_2_	2.09–2.25*	36.8, CH_2_	1.35–1.42,m	40.4, CH_2_	1.96, t (7.5)
				1.00, m		
**16**	27.7, CH_2_	2.09–2.25*	23.0, CH_2_	2.09–2.14, m	27.4, CH_2_	2.07*
				1.96–2.01, m		
**17**	125.2, CH	5.16, t (6.6)	126.1, CH	4.98, t (7.0)	125.1, CH	5.10, t (7.1)
**18**	131.9, C		130.9, C		131.6, C	
**19**	17.7, CH_3_	1.60, s	17.6, CH_3_	1.56, s	17.7, CH_3_	1.58, s
**20**	25.8, CH_3_	1.66, br. s	25.8, CH_3_	1.59, s	25.8, CH_3_	1.65, s
**21**	109.7, CH_2_	4.64, br. s	27.3, CH_3_	1.13, s	15.9, CH_3_	1.62, s
		4.28, br. s				
**22**	29.1, CH_3_	1.28, s	29.0, CH_3_	1.26, s	27.5, CH_3_	1.20. s
**4-O** ***H***		7.96, s		7.91, s		8.05, s
**6-O** ***H***						8.05, s
**10-O** ***H***						3.54, s
**14-O** ***H***				3.30, s		

^a^^1^H NMR spectra were measured at 400 MHz, and ^13^C NMR spectra at 100 MHz

^b^^1^H NMR spectra was measured at 500 MHz, and ^13^C NMR spectra at 125 MHz

* Signals were overlapped

### Albatrelin H (**8**)

Colorless oil; [α]_D_^22^ −9.1 (*c* 0.21, MeOH); UV *λ*_max_ (MeOH) (log *ε*) 284 (2.63) nm; IR (KBr) *ν*_max_ 3441, 2968, 2929, 2872, 2855, 1615, 1595, 1452, 1145 cm^−1^; ^1^H and ^13^C NMR spectroscopic data, see Table 3; EI-MS *m/z*: 344 [M]^+^, 175, 137; HR-EI-MS *m/z*: 344.2345 [M]^+^ (calcd for C_22_H_32_O_3_, 344.2351).

### (*S*)-10-hydroxygrifolin (**9**)

Colorless oil; [α]_D_^22^ −8.7 (*c* 0.20, MeOH); UV *λ*_max_ (MeOH) (log *ε*) 276 (2.50) nm; IR (KBr) *ν*_max_ 3441, 2967, 2925, 2856, 1628, 1598, 1452, 1381, 1050 cm^−1^; ^1^H and ^13^C NMR spectroscopic data, see Table 3; EI-MS *m/z*: 346 [M]^+^, 328 [M–H_2_O]^+^, 175, 137; HR-EI-MS *m/z*: 346.2505 [M]^+^ (calcd for C_22_H_34_O_3_, 346.2508).

### Preparation of **6a**

To a solution of compound **6** (2.3 mg, 6.35 *μ*mol) in DMF (2 mL) were added 2,2-dimethoxypropane (1.3 mg, 12.7 *μ*mol) and *p*-toluenesulfonic acid monohydrate (0.6 mg, 3.18 *μ*mol), and the mixture was stirred for 30 min at room temperature. The reaction mixture was added into water, and then extracted by EtOAc for three times. The organic layer was evaporated and the residue was chromatographed on a column of silica gel eluting with petroleum ether-acetone 40:1 to yield **6a** (2.1 mg).

### Cytotoxic assay

The following human tumor cell lines were used: HL-60, SMMC-7712, A-549, MCF-7, and SW480. All the cells were cultured in RMPI-1640 or DMEM medium (Hyclone, Logan, UT), supplemented with 10 % fetal bovine serum (Hyclone) at 37 °C in a humidified atmosphere with 5 % CO_2_. Cell viability was assessed by conducting colorimetric measurements of the amount of insoluble formazan formed in living cells based on the reduction of 3-(4,5-dimethylthiazol-2-yl)-2,5-diphenyltetrazolium bromide (MTT) (Sigma, St. Louis, MO). Briefly, 100 *μ*L of adherent cells were seeded into each well of a 96-well cell culture plate and allowed to adhere for 12 h before drug addition, while suspended cells were seeded just before drug addition, both with an initial density of 1 × 10^5^ cells/mL in 100 *μ*L of medium. Each tumor cell line was exposed to the test compounds at various concentrations in triplicate for 48 h, with DDP and toxal as positive controls. After the incubation, MTT (100 *μ*g) was added to each well, and the incubation continued for 4 h at 37 °C. The cells lysed with 200 *μ*L SDS after removal of 100 *μ*L of medium. The optical density of lysate was measured at 595 nm in a 96-well microtiter plate reader (Bio-Rad 680). The IC_50_ value of each compound was calculated by Reed and Muench’s method [17].

## Electronic Supplementary Material

Below is the link to the electronic supplementary material.

Supplementary material 1 (DOC 8005 kb)
