# Supplementary material for: Nine New Farnesylphenols from the Basidiomycete Albatrellus Caeruleoporus
Source: Nat Prod Bioprospect. 2014 Apr 23;4(2):119–28. doi: 10.1007/s13659-014-0015-5 (PMC4004861; doi:10.1007/s13659-014-0015-5)

**Farnesylphenols from the basidiomycete *Albatrellus caeruleoporus***

Liang-Yan Liu ^a,b^, Zheng-Hui Li ^a^, Gang-Qiang Wang ^a^, Kun Wei ^a^, Ze-Jun Dong ^a^, Tao Feng^a^, Gen-Tao Li ^a^, Yan Li ^a^, and Ji-Kai Liu ^a^,*

^a^ State Key Laboratory of Phytochemistry and Plant Resources in West China, Kunming Institute of Botany, Chinese Academy of Sciences, Kunming, 650201, Yunnan, PR China, and ^b^ Graduate University of the Chinese Academy of Sciences, Beijing, 100049, PR China

*Corresponding author. Tel.: +86-871-5216327; Fax: +86-871-5212285; E-mail address: [jkliu@mial.kib.ac.cn](mailto:jkliu@mial.kib.ac.cn) (J.-K. Liu).

**Content table**

1D and 2D NMR spectra for (*S*)-17-hydroxy-18,20-ene-neogrifolin (**1**)……………………………....S3

1D and 2D NMR spectra for (*S*)-18,19-dihydroxyneogrifolin (**2**)……..……..…………………….…..S5

1D and 2D NMR spectra for (*S*)-9-hydroxy-10,22-ene-neogrifolin (**3**)…… ………………………..S8

1D and 2D NMR spectra for (9*S*,10*R*)-6,10-epoxy-9-hydroxyneo grifolin (**4**)………………………..S10

1D and 2D NMR spectra for (9*S*,10*R*)-6,9-epoxy-10-hydroxyneogrifolin (**5**) ………………………..S13

1D and 2D NMR spectra for (−)-13,14-dihydr oxyneogrifolin (**6**)………………..…………………..S16

1D and 2D NMR spectra for albatrelin G (**7**)……………………………………………………….….S19

1D and 2D NMR spectra for albatrelin H (**8**)…………………………………………………………..S22

1D and 2D NMR spectra for (*S*)-10-hydroxygrifolin (**9**)………………………………………………S25

^1^H and ROESY NMR spectra for **6a**……………………………………………………………………S27

^1^H NMR spectrum for (*S*)-17-hydroxy-18,20-ene-neogrifolin (**1**)


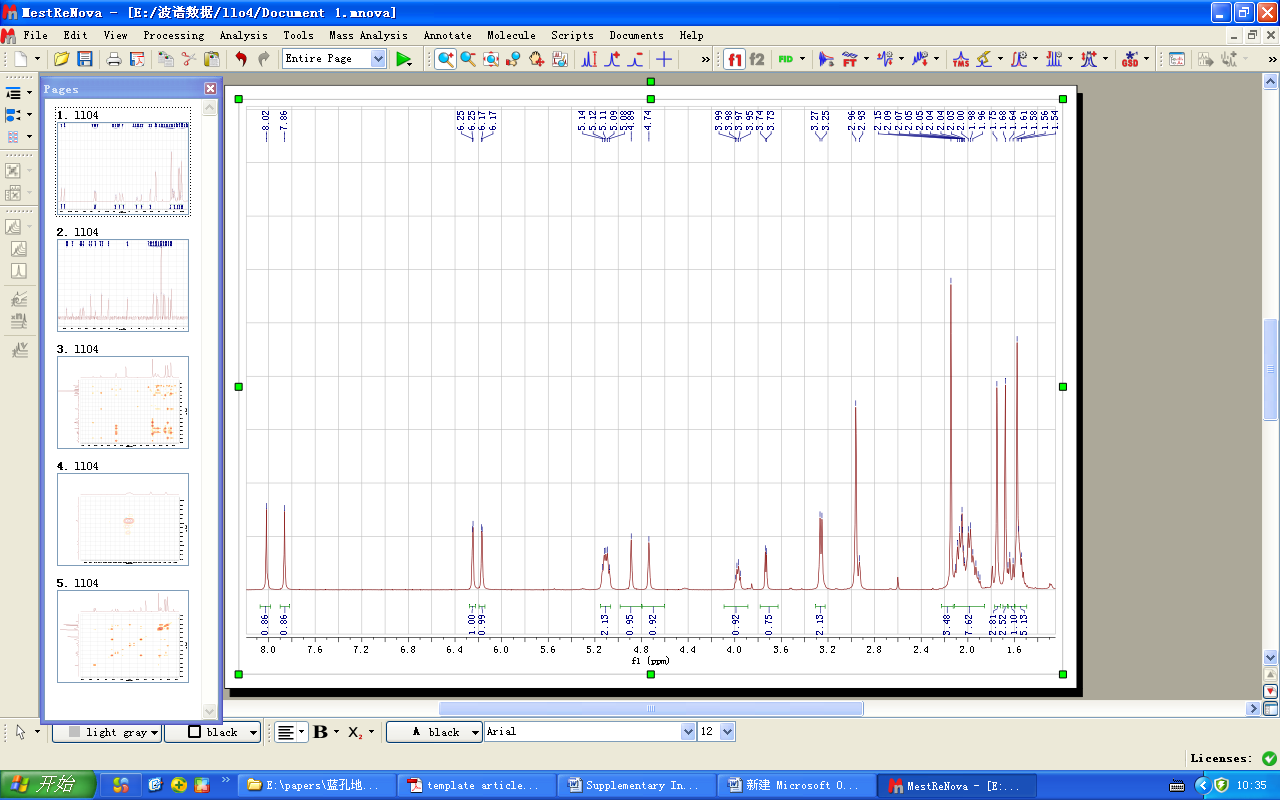


^13^C NMR spectrum for (*S*)-17-hydroxy-18,20-ene-neogrifolin (**1**)


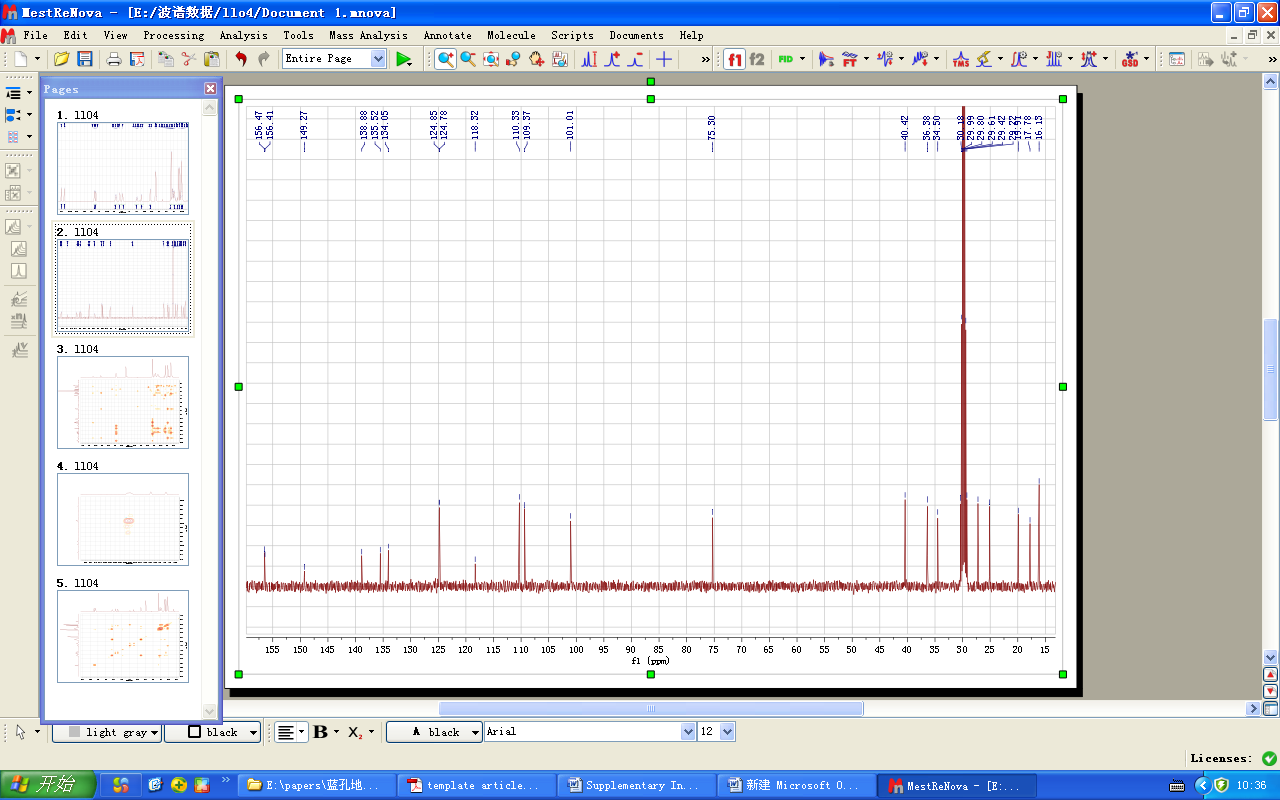


HSQC spectrum for (*S*)-17-hydroxy-18,20-ene-neogrifolin (**1**)


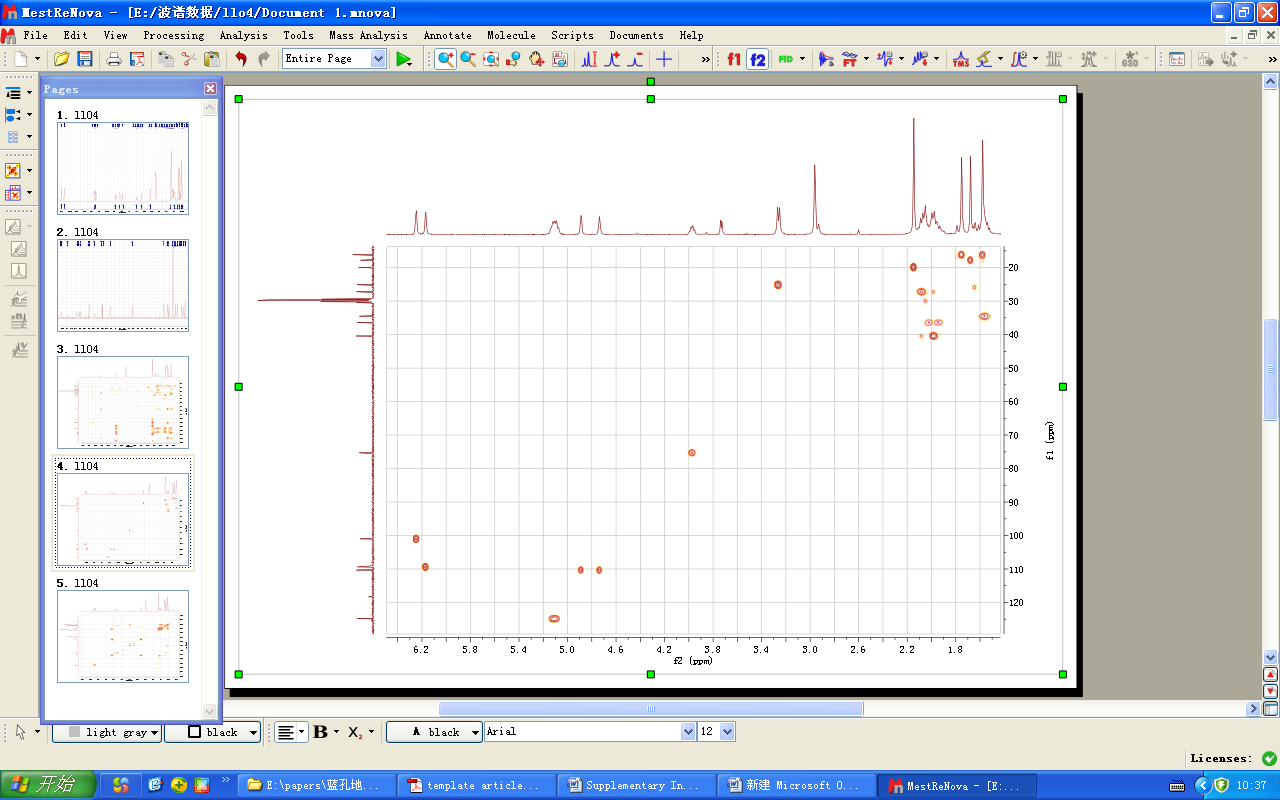


HMBC spectrum for (*S*)-17-hydroxy-18,20-ene-neogrifolin (**1**)


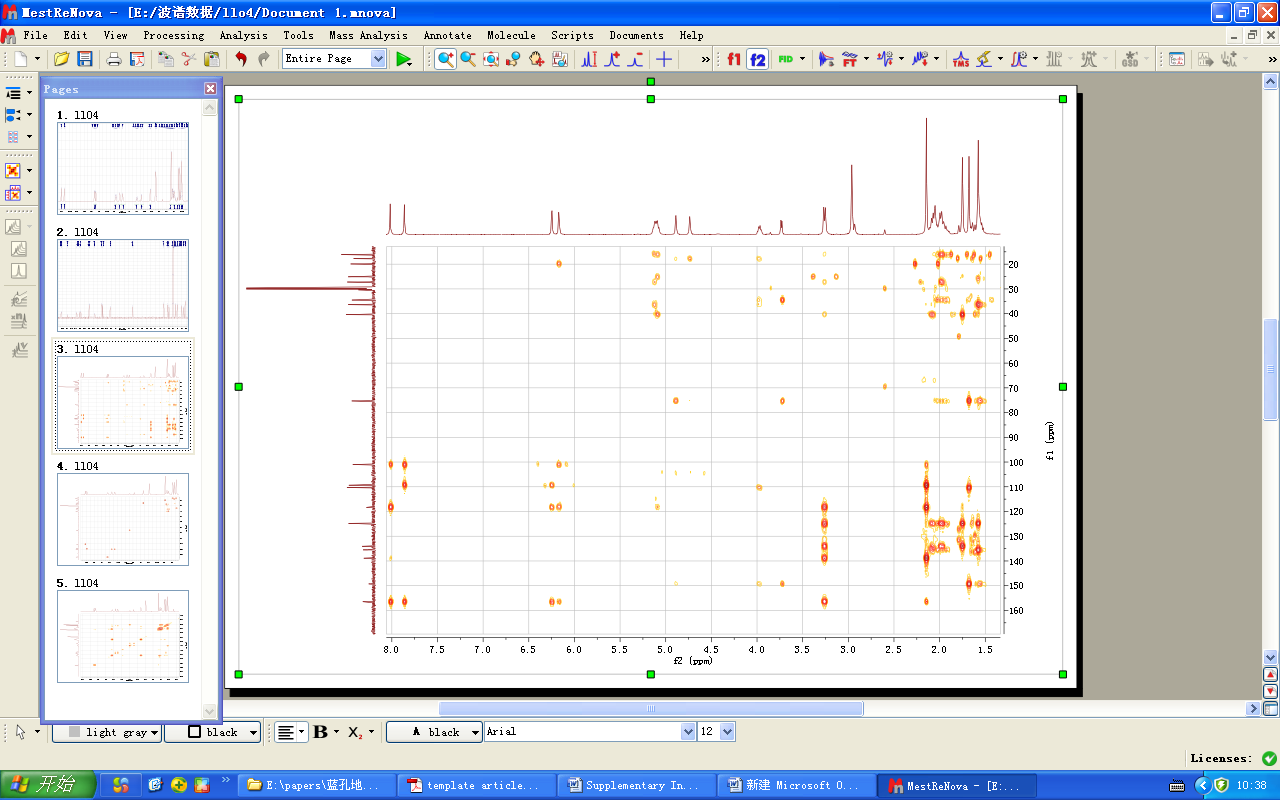


COSY spectrum for (*S*)-17-hydroxy-18,20-ene-neogrifolin (**1**)


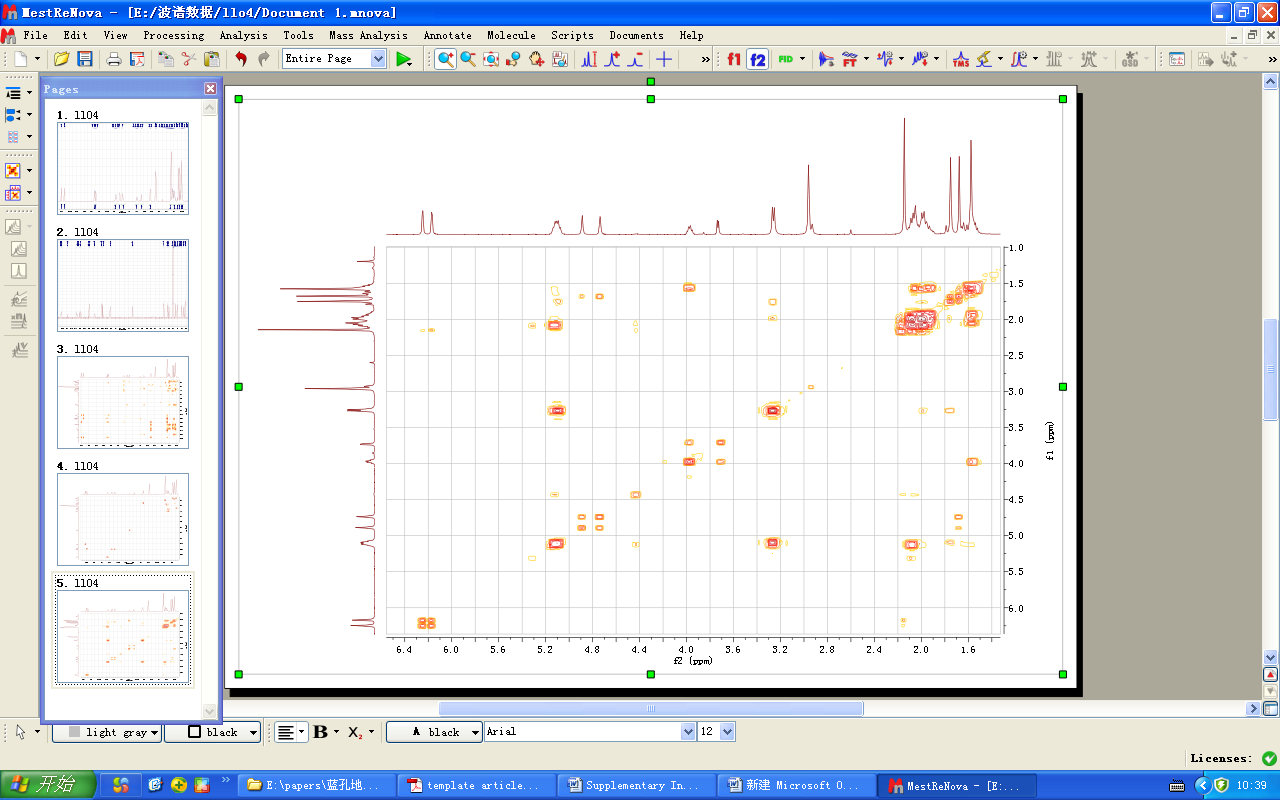


^1^H NMR spectrum for (*S*)-18,19-dihydroxyneogrifolin (**2**)


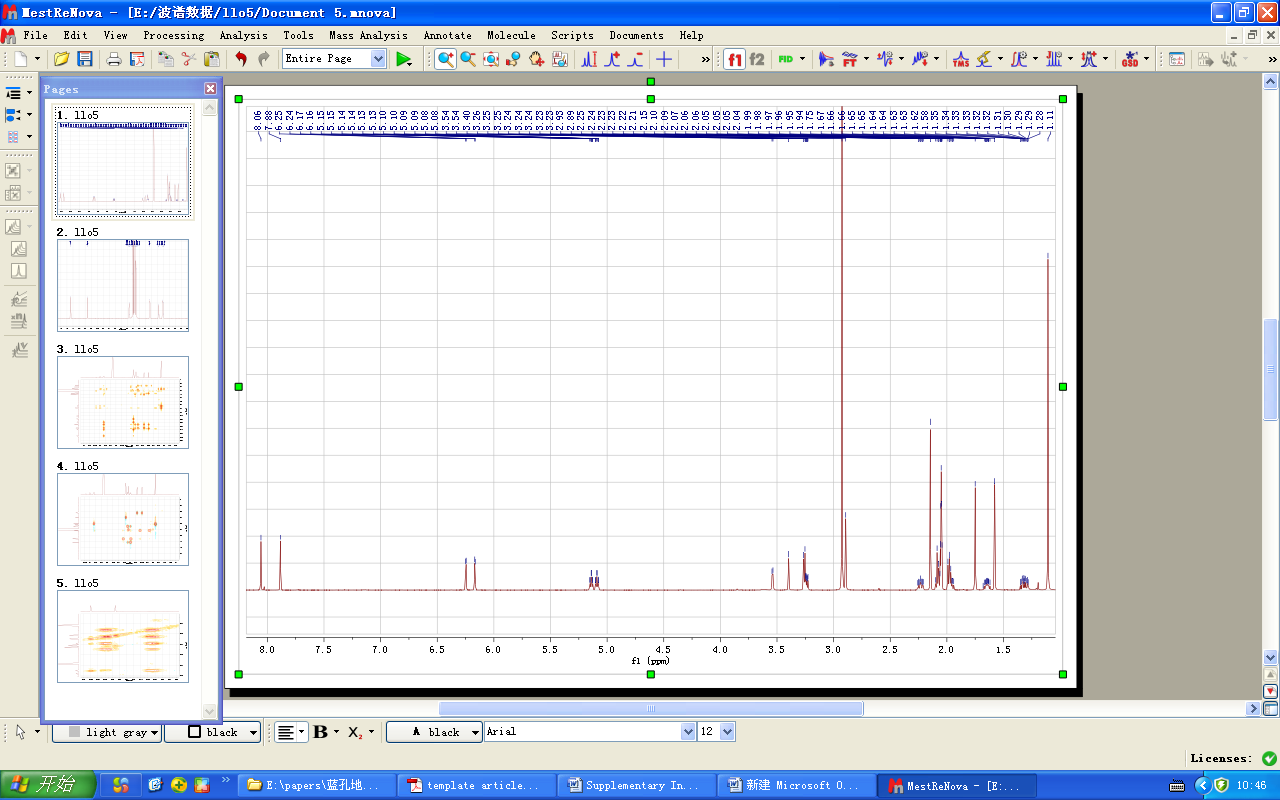


^13^C NMR spectrum for (*S*)-18,19-dihydroxyneogrifolin (**2**)


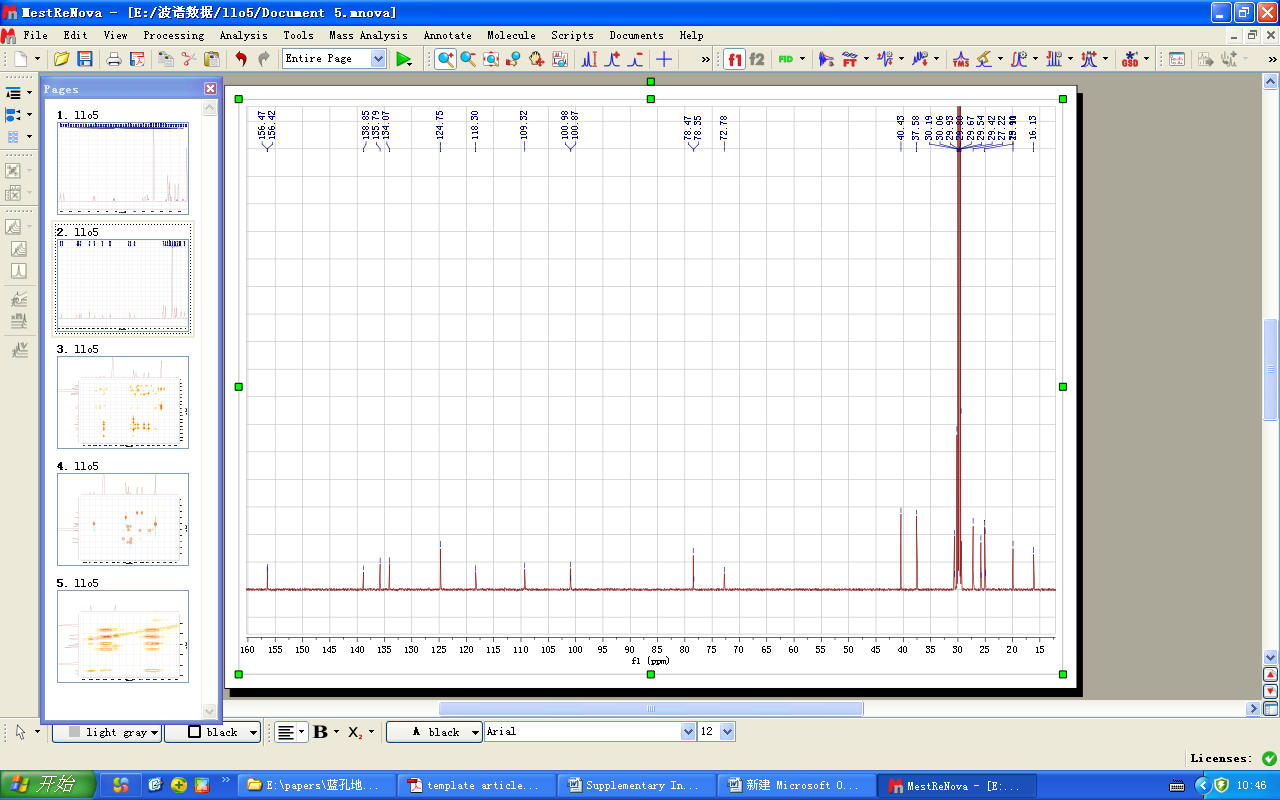


HSQC spectrum for (*S*)-18,19-dihydroxyneogrifolin (**2**)


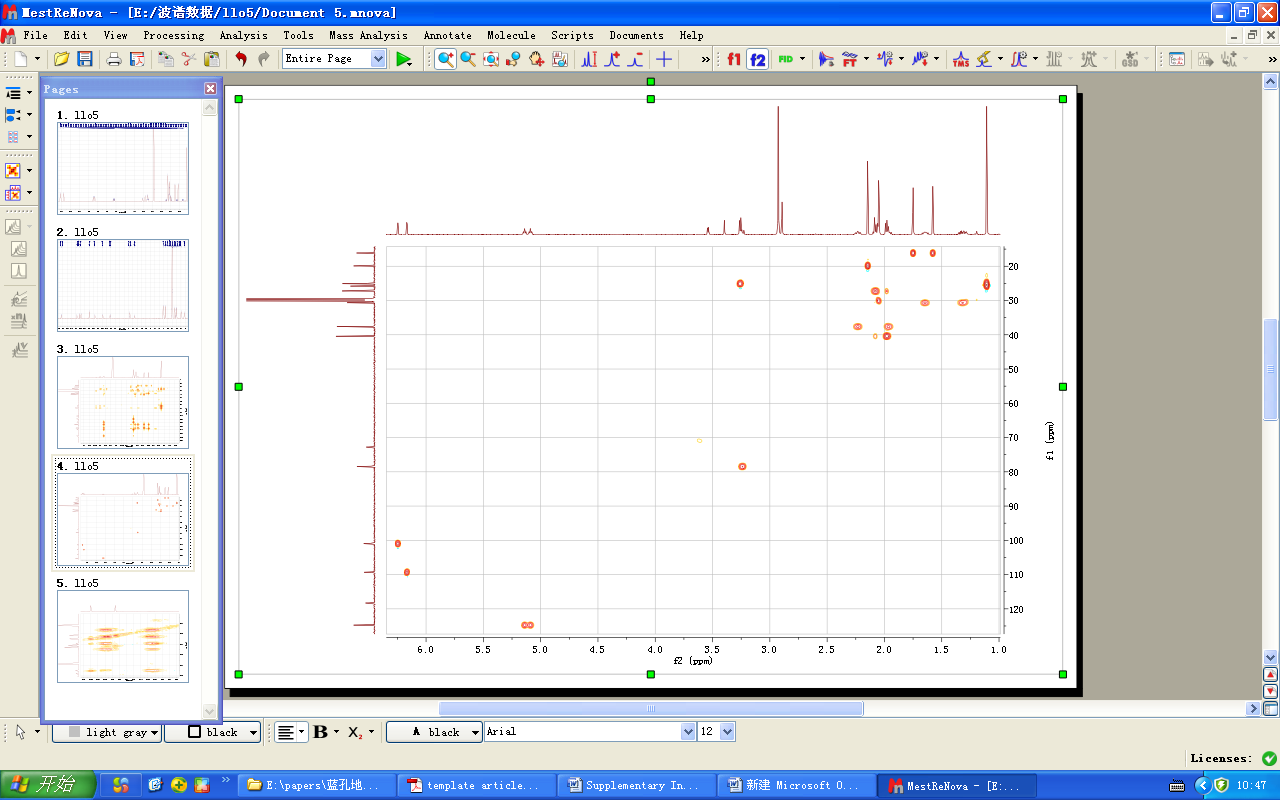


HMBC spectrum for (*S*)-18,19-dihydroxyneogrifolin (**2**)


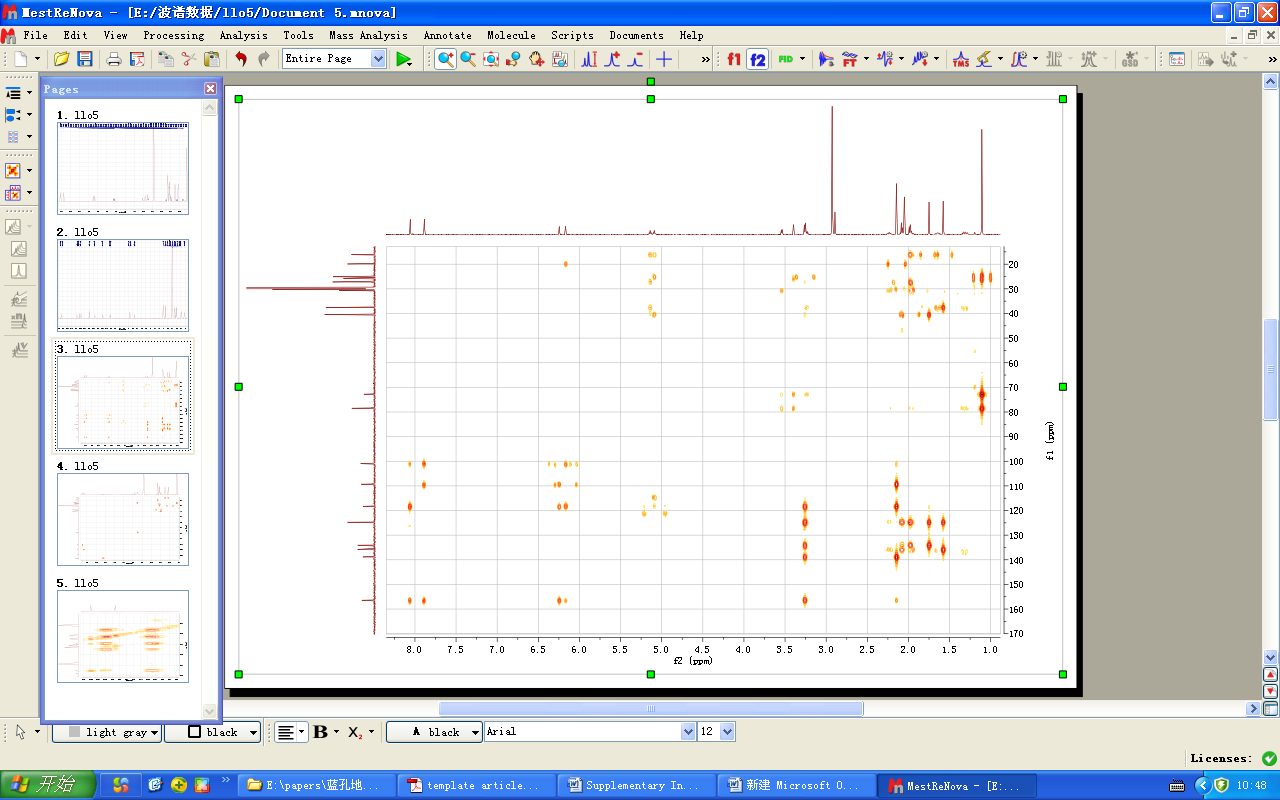


COSY spectrum for (*S*)-18,19-dihydroxyneogrifolin (**2**)


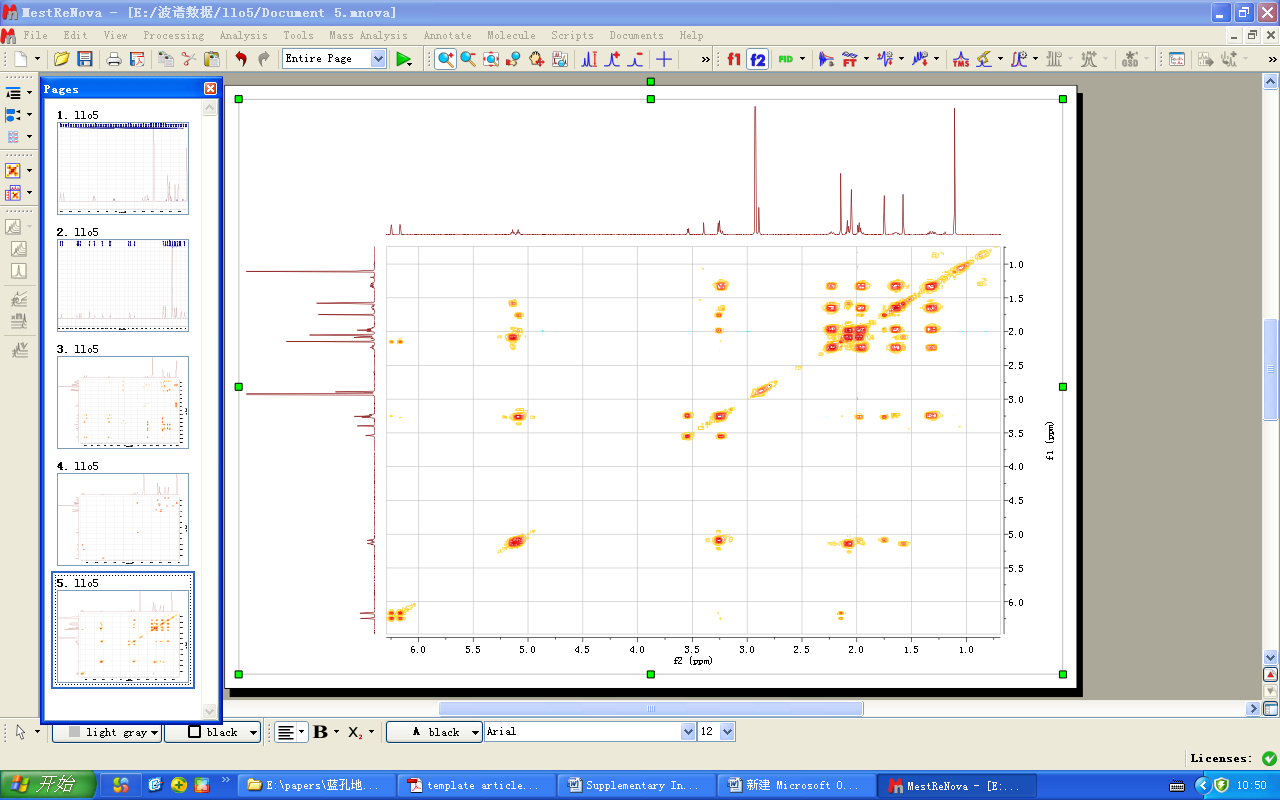


^1^H NMR spectrum for (*S*)-9-hydroxy-10,22-ene-neogrifolin (**3**)


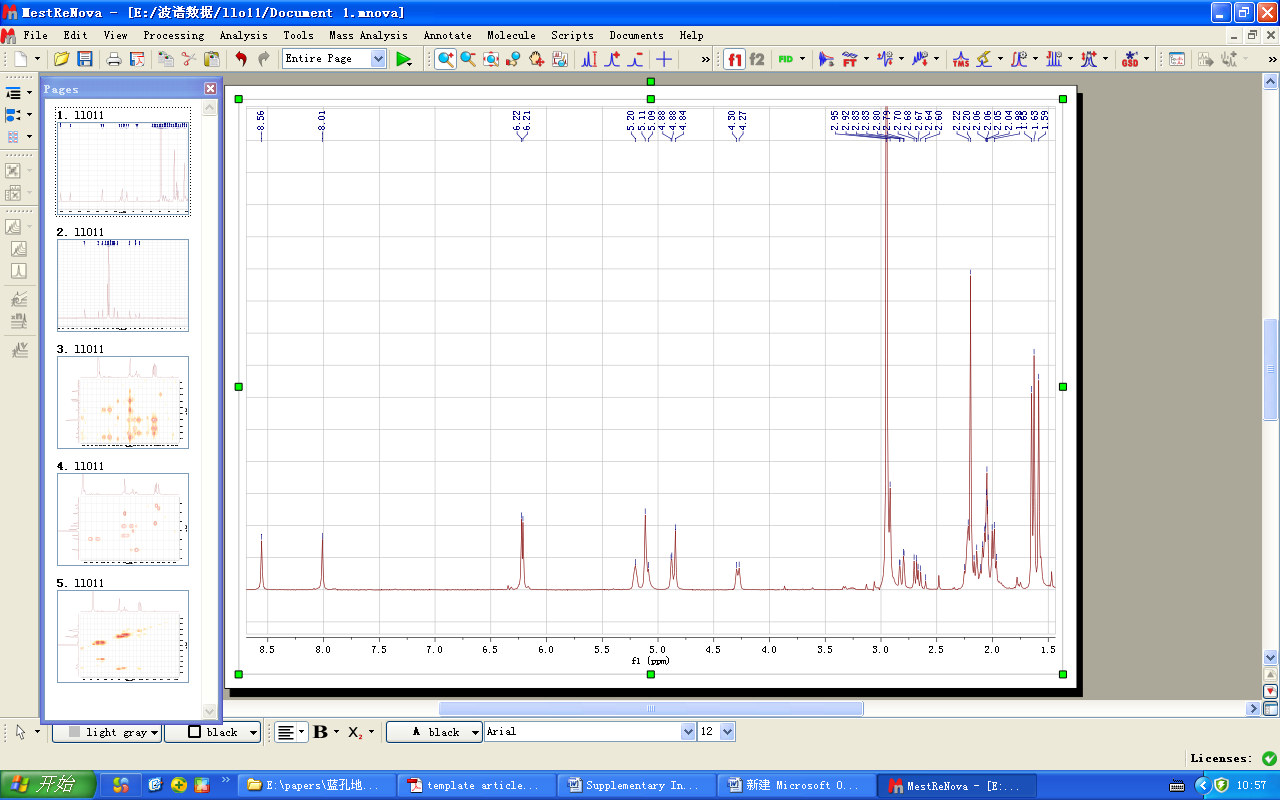


^13^C NMR spectrum for (*S*)-9-hydroxy-10,22-ene-neogrifolin (**3**)


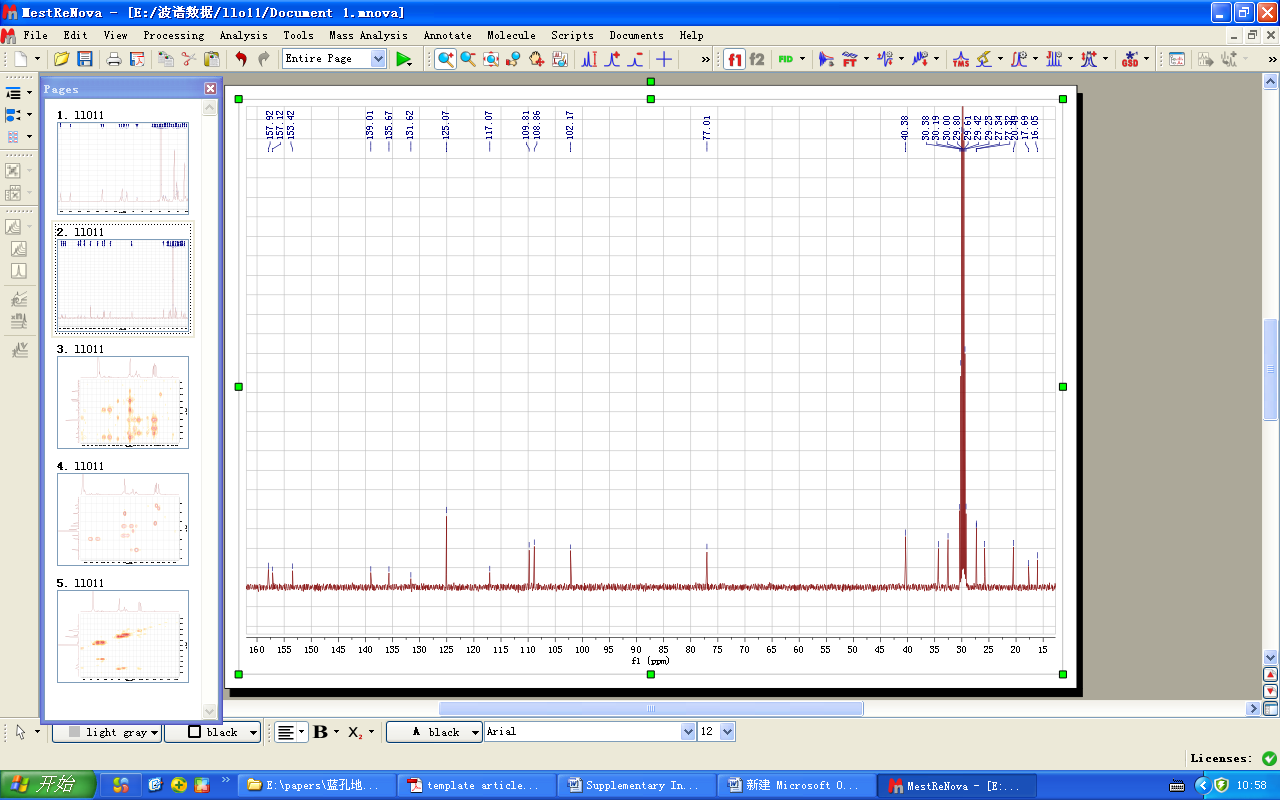


HSQC spectrum for (*S*)-9-hydroxy-10,22-ene-neogrifolin (**3**)


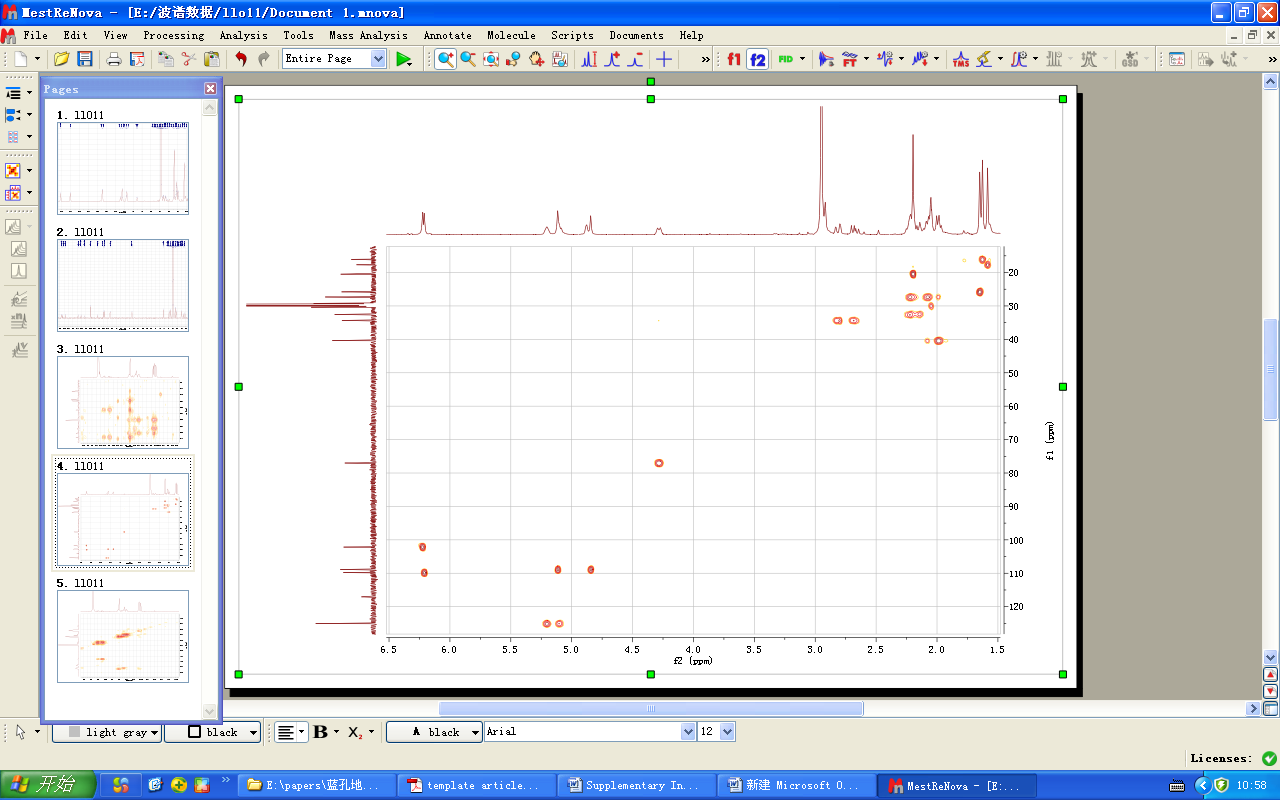


HMBC spectrum for (*S*)-9-hydroxy-10,22-ene-neogrifolin (**3**)


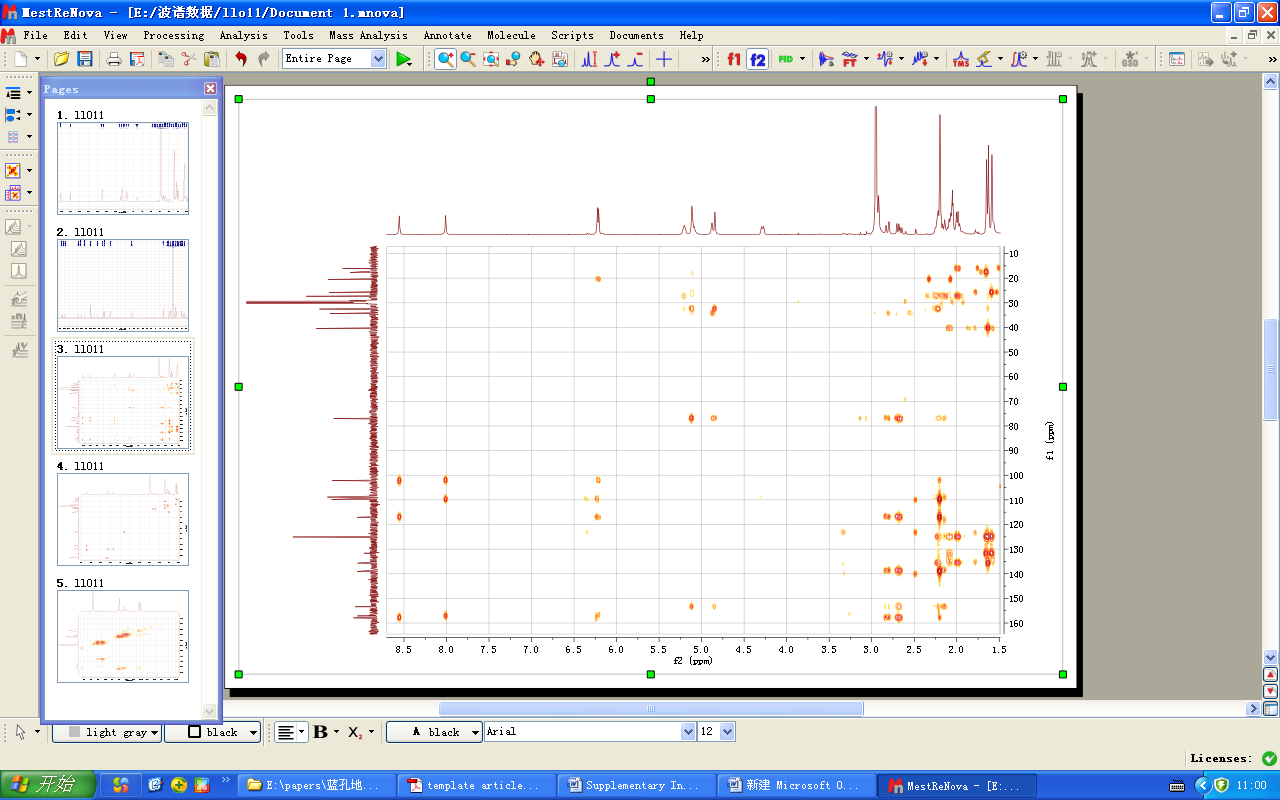


COSY spectrum for (*S*)-9-hydroxy-10,22-ene-neogrifolin (**3**)


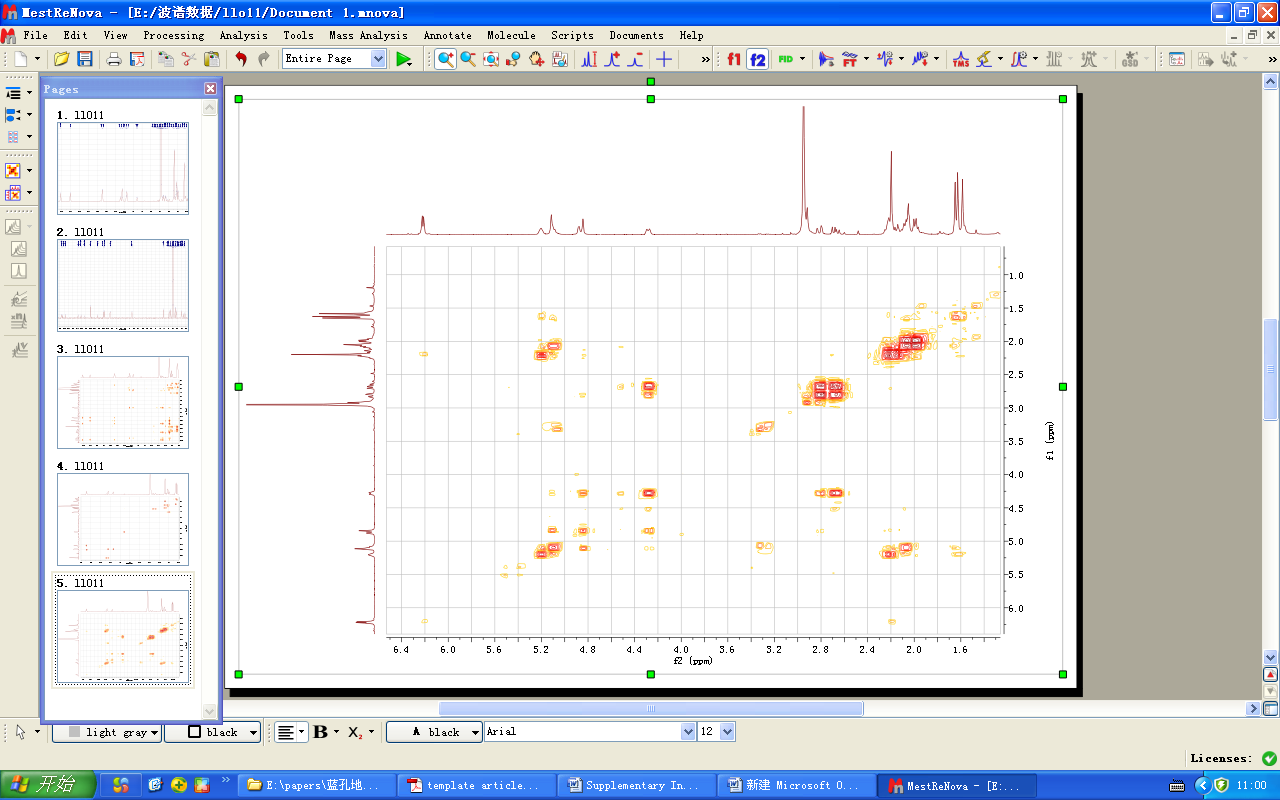


^1^H NMR spectrum for (9*S*,10*R*)-6,10-epoxy-9-hydroxyneo grifolin (**4**)


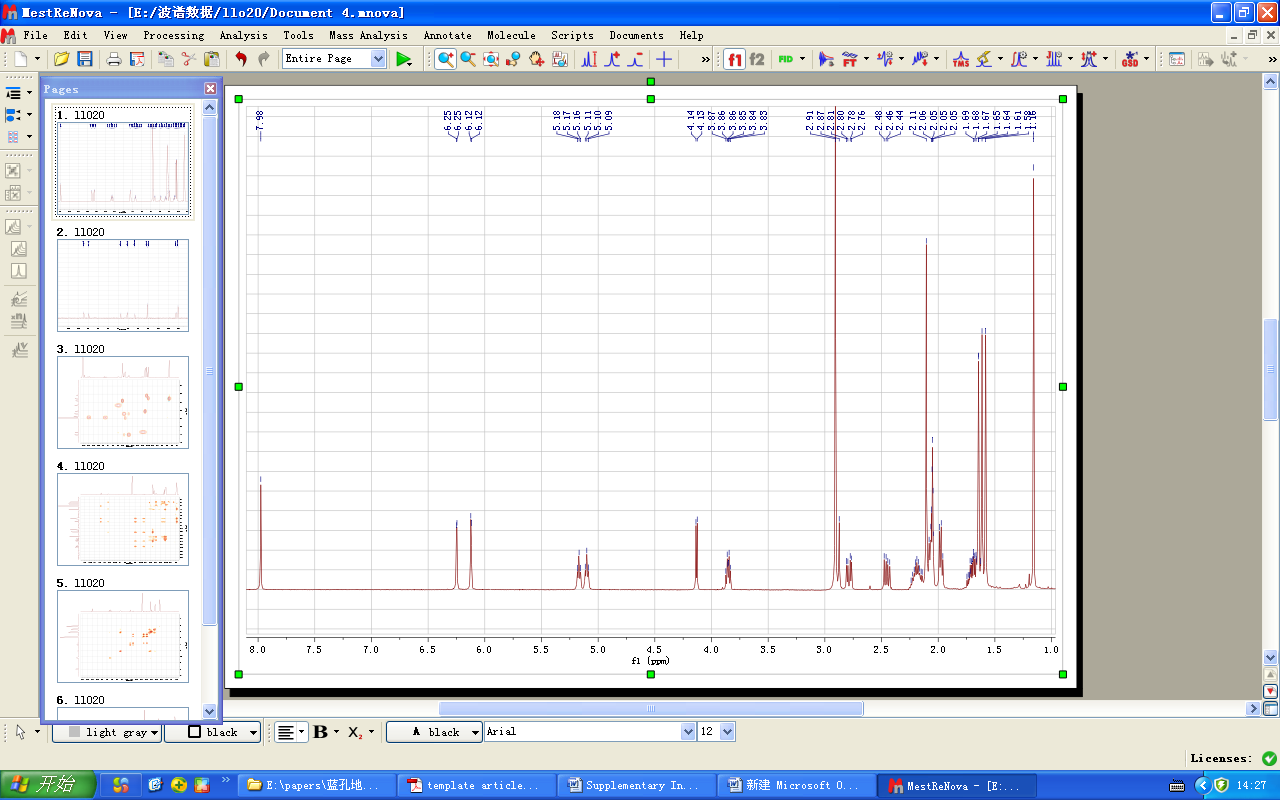


^13^C NMR spectrum for (9*S*,10*R*)-6,10-epoxy-9-hydroxyneo grifolin (**4**)


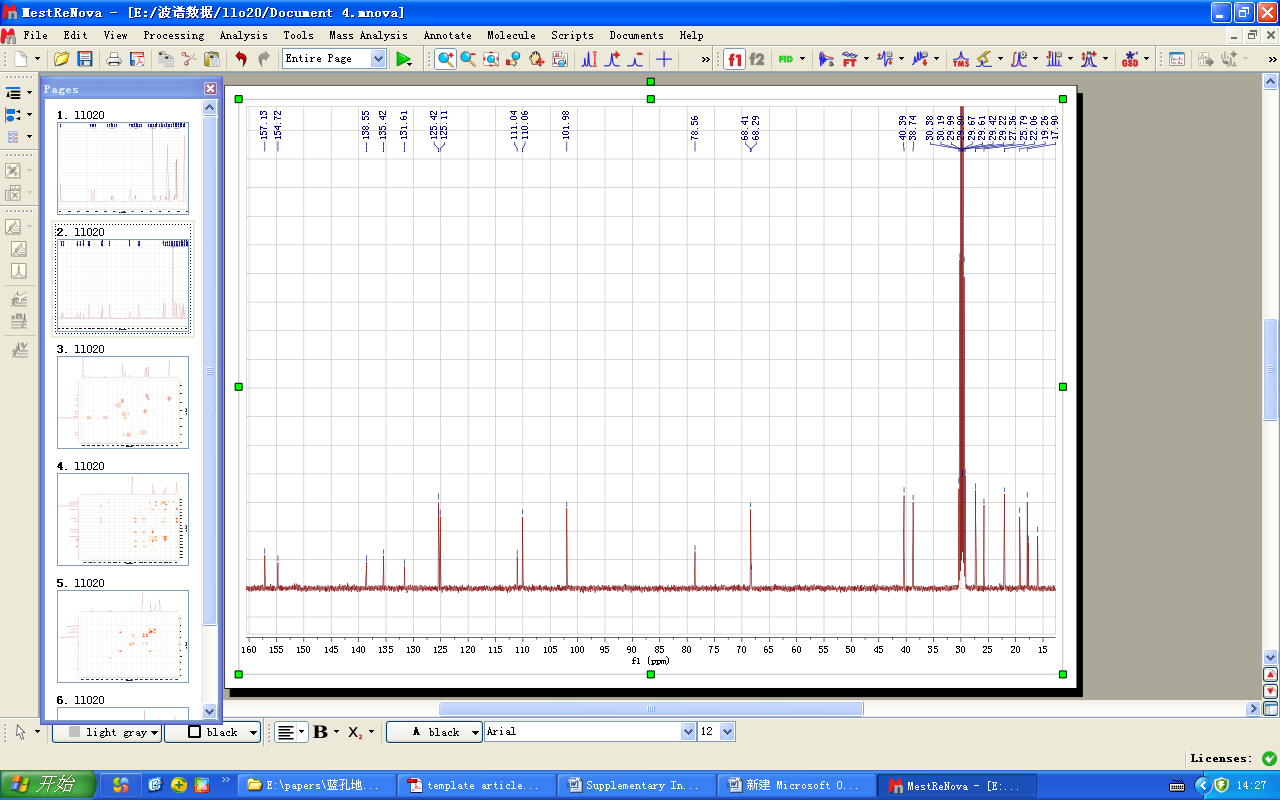


HSQC spectrum for (9*S*,10*R*)-6,10-epoxy-9-hydroxyneo grifolin (**4**)


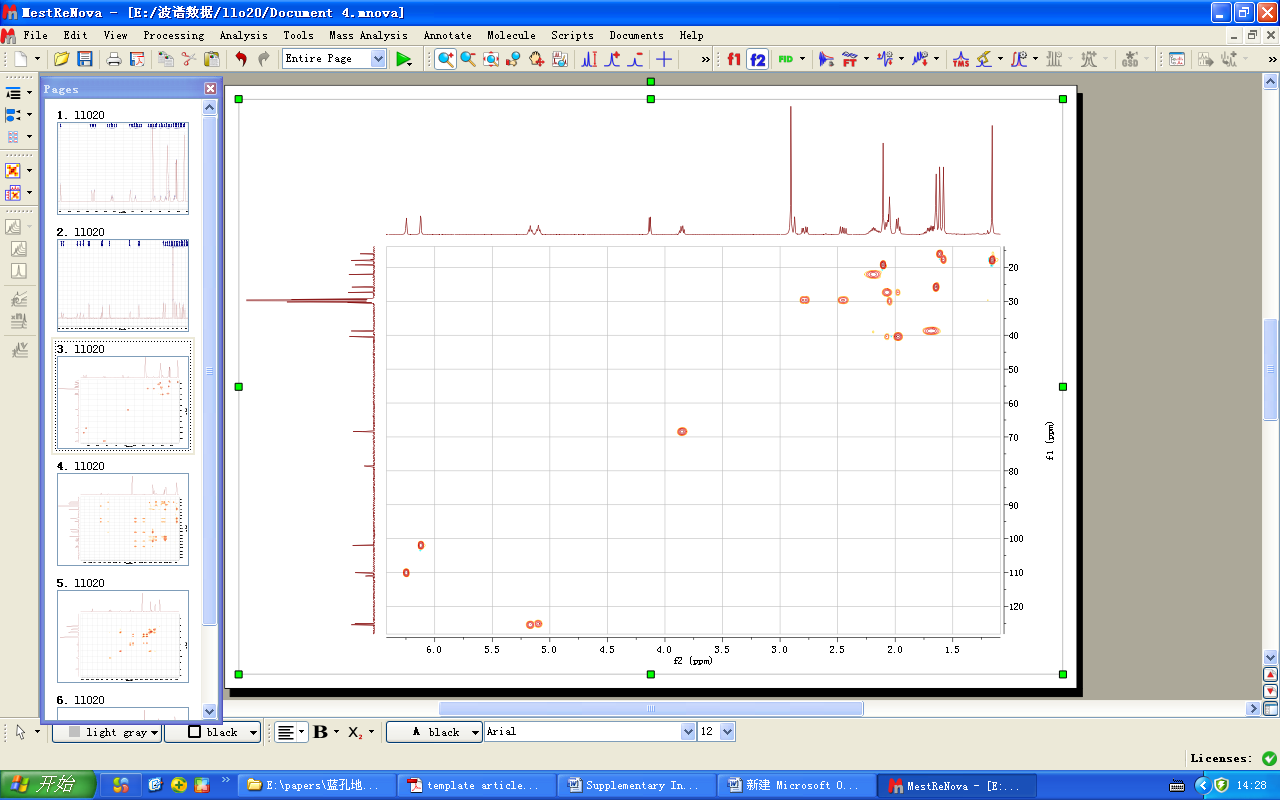


HMBC spectrum for (9*S*,10*R*)-6,10-epoxy-9-hydroxyneo grifolin (**4**)


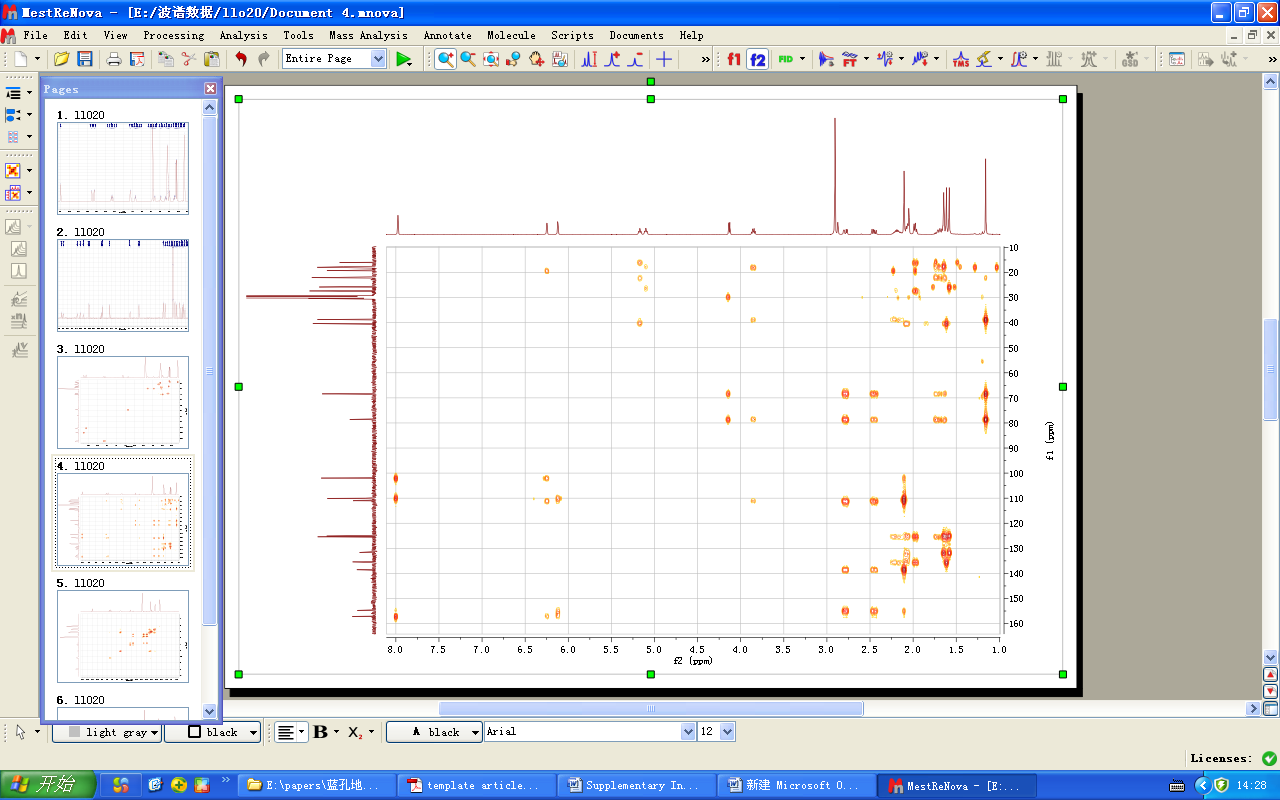


COSY spectrum for (9*S*,10*R*)-6,10-epoxy-9-hydroxyneo grifolin (**4**)


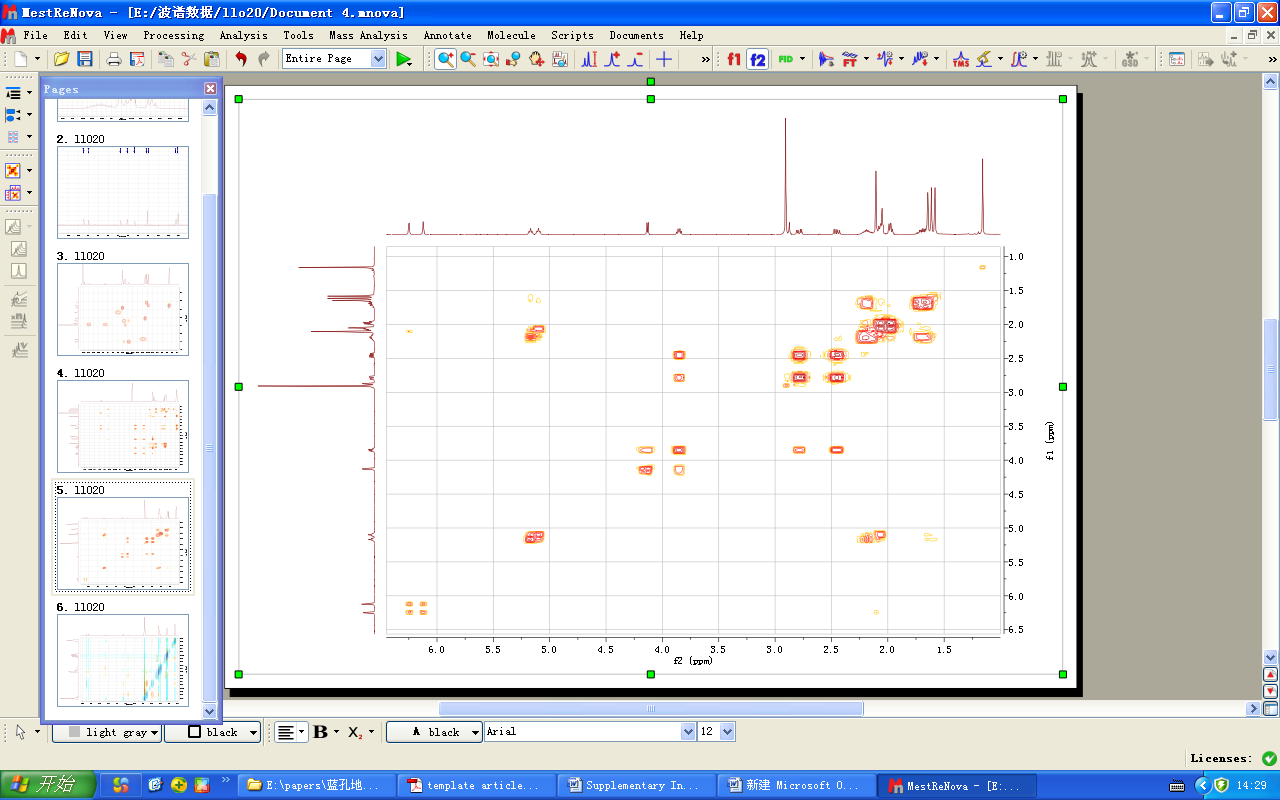


ROESY spectrum for (9*S*,10*R*)-6,10-epoxy-9-hydroxyneo grifolin (**4**)


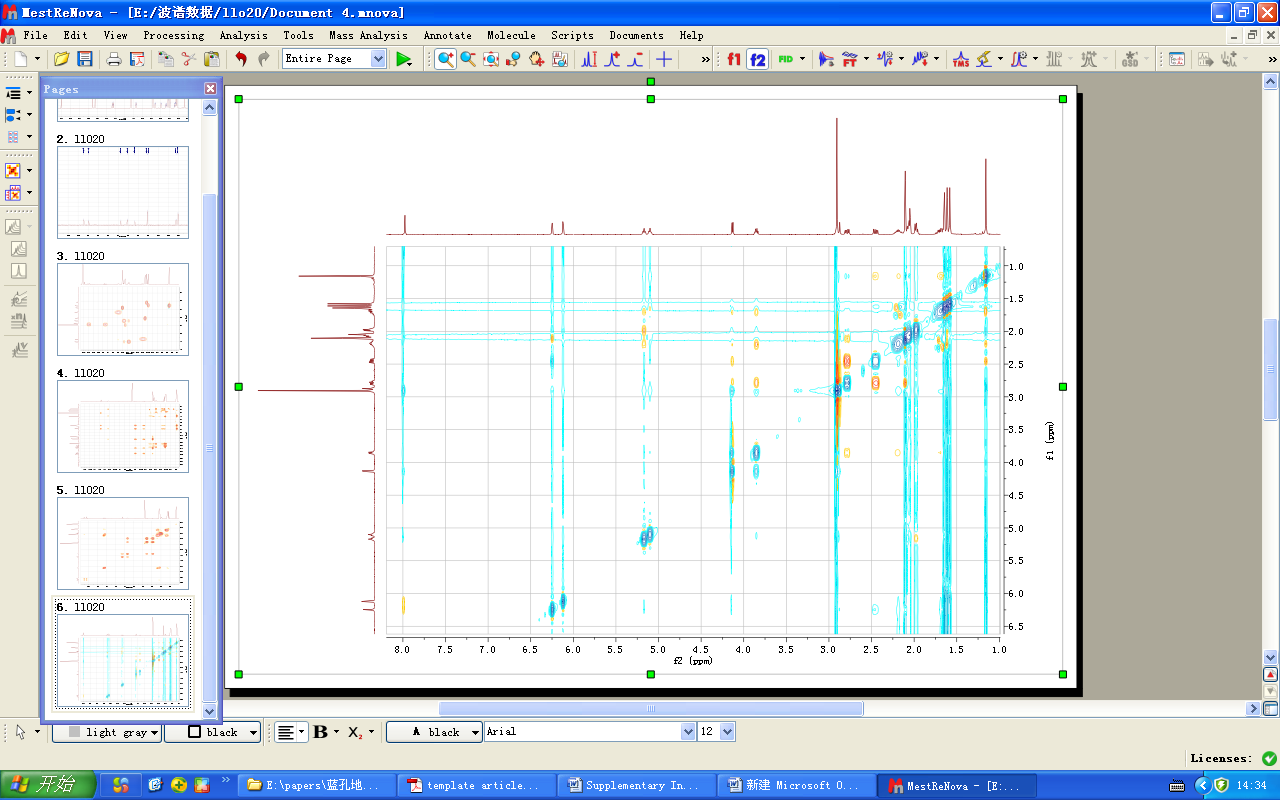


^1^H NMR spectrum for (9*S*,10*R*)-6,9-epoxy-10-hydroxyneogrifolin (**5**)


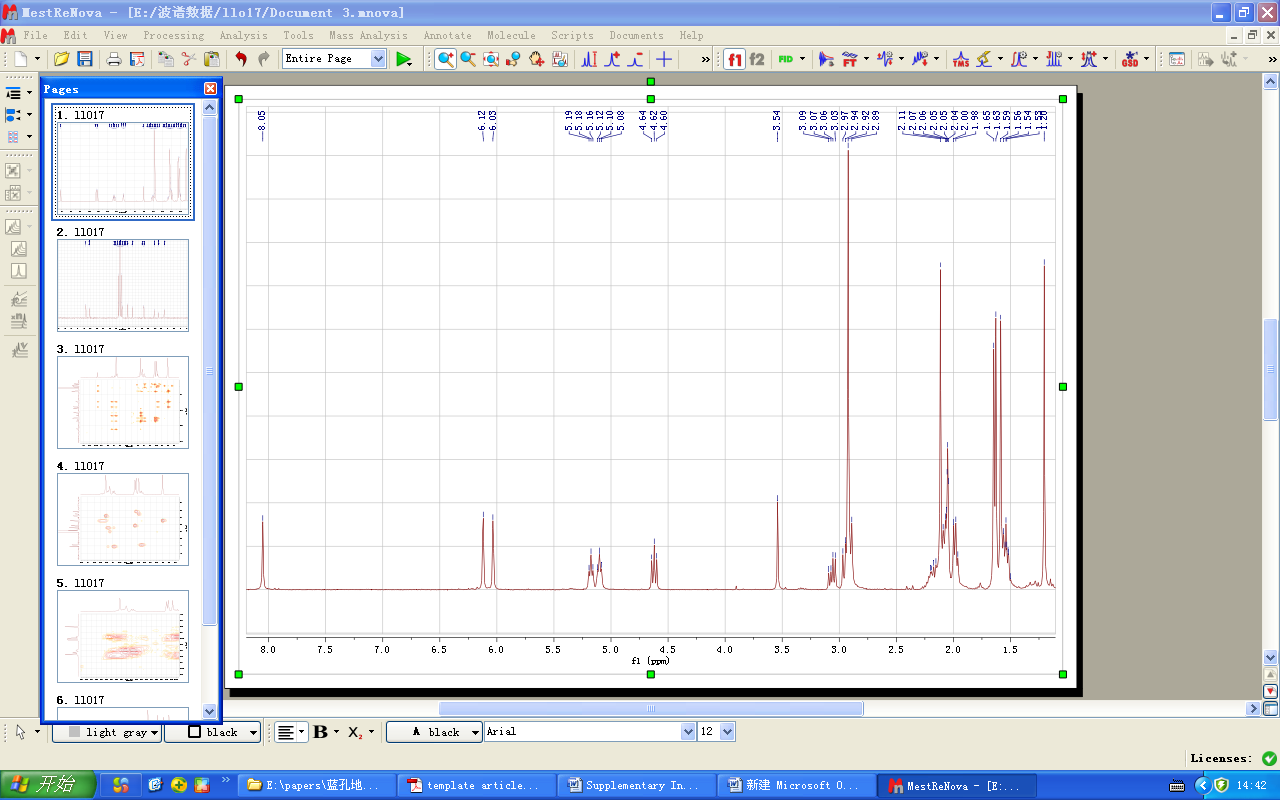


^13^C NMR spectrum for (9*S*,10*R*)-6,9-epoxy-10-hydroxyneogrifolin (**5**)


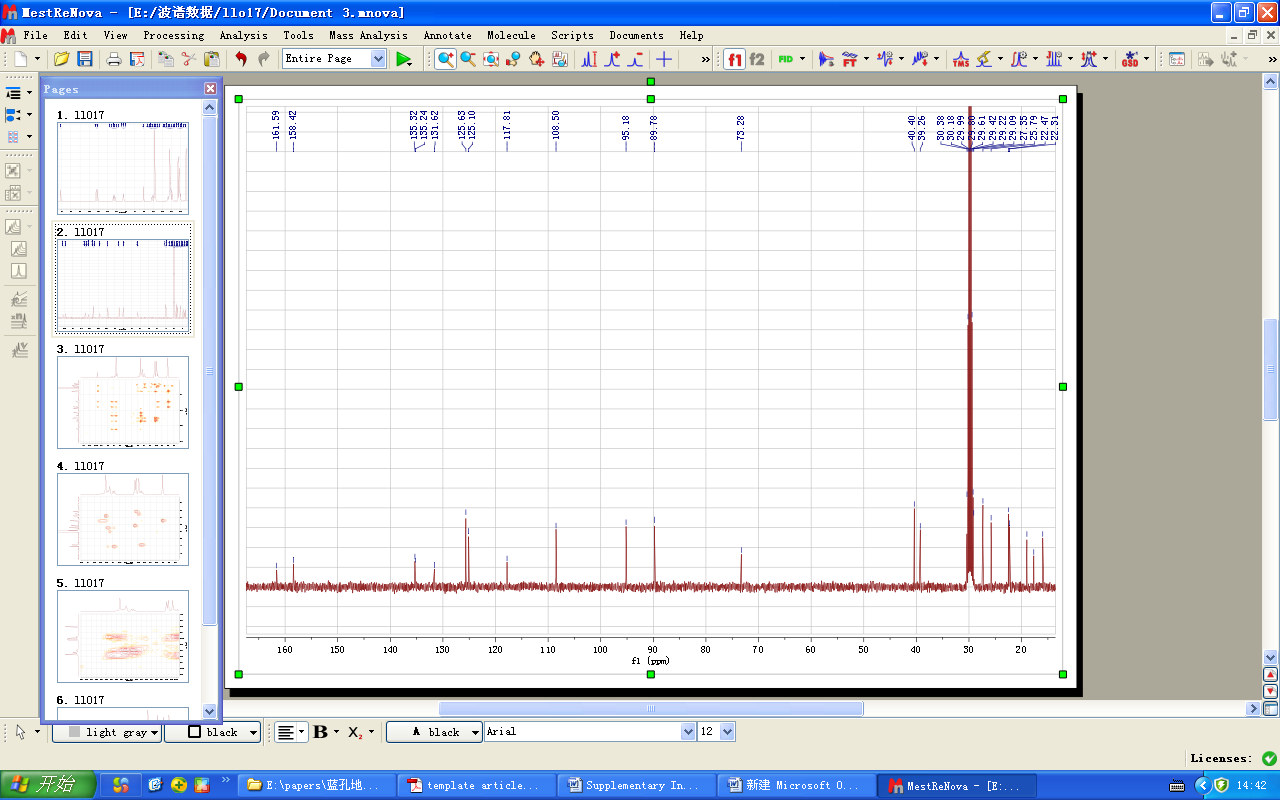


HSQC spectrum for (9*S*,10*R*)-6,9-epoxy-10-hydroxyneogrifolin (**5**)


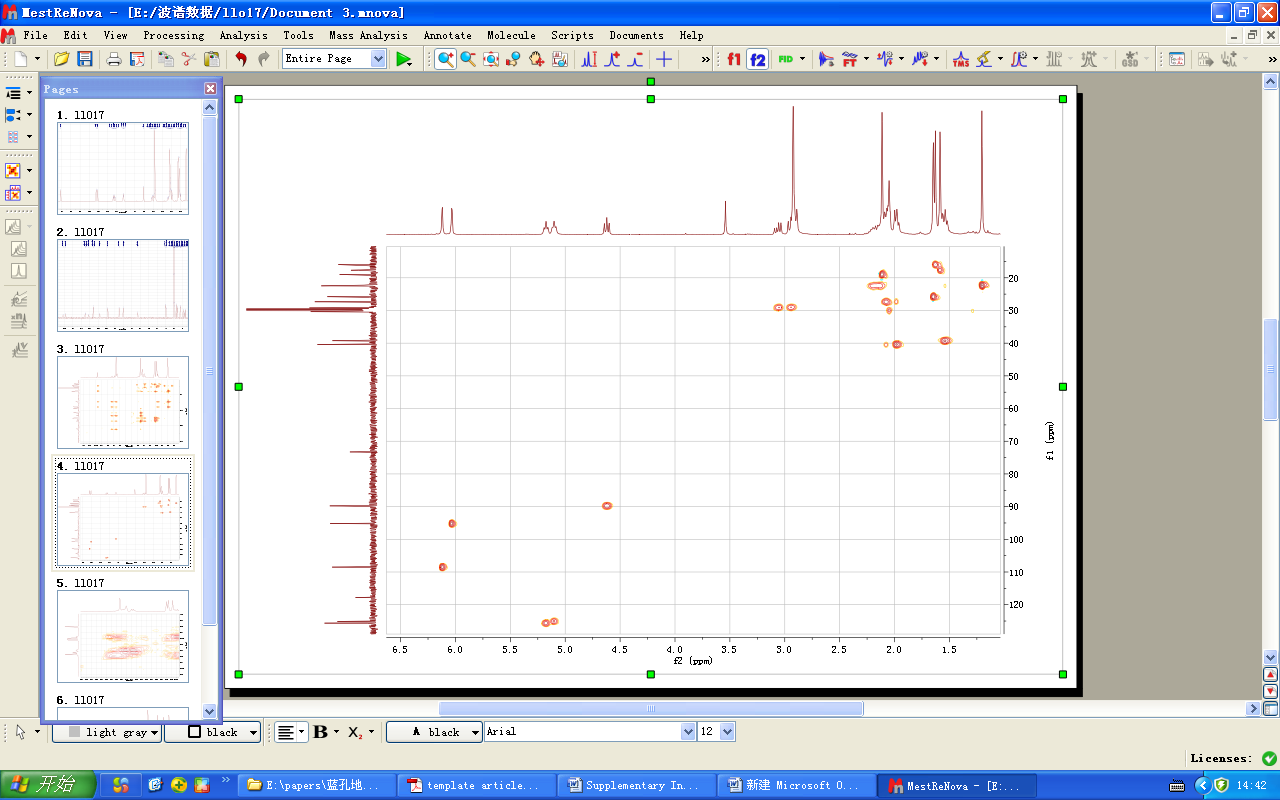


HMBC spectrum for (9*S*,10*R*)-6,9-epoxy-10-hydroxyneogrifolin (**5**)


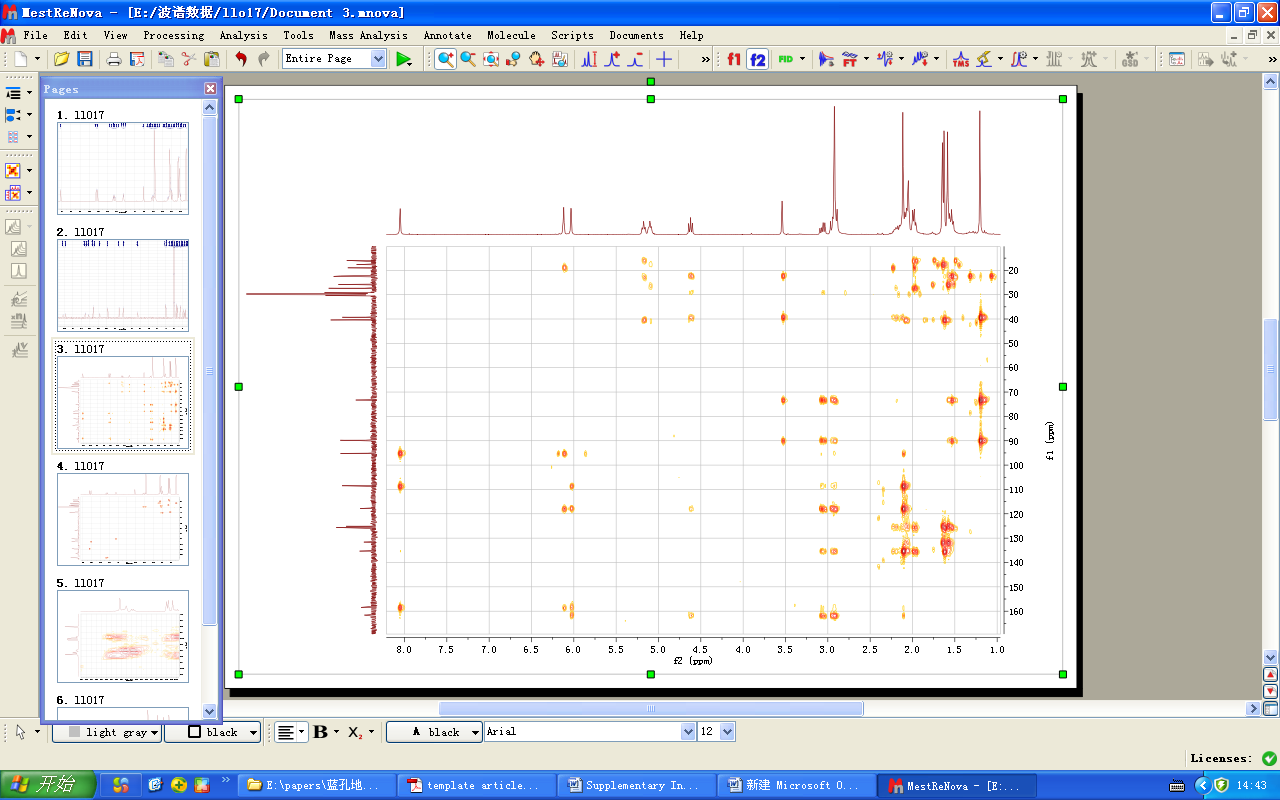


COSY spectrum for (9*S*,10*R*)-6,9-epoxy-10-hydroxyneogrifolin (**5**)


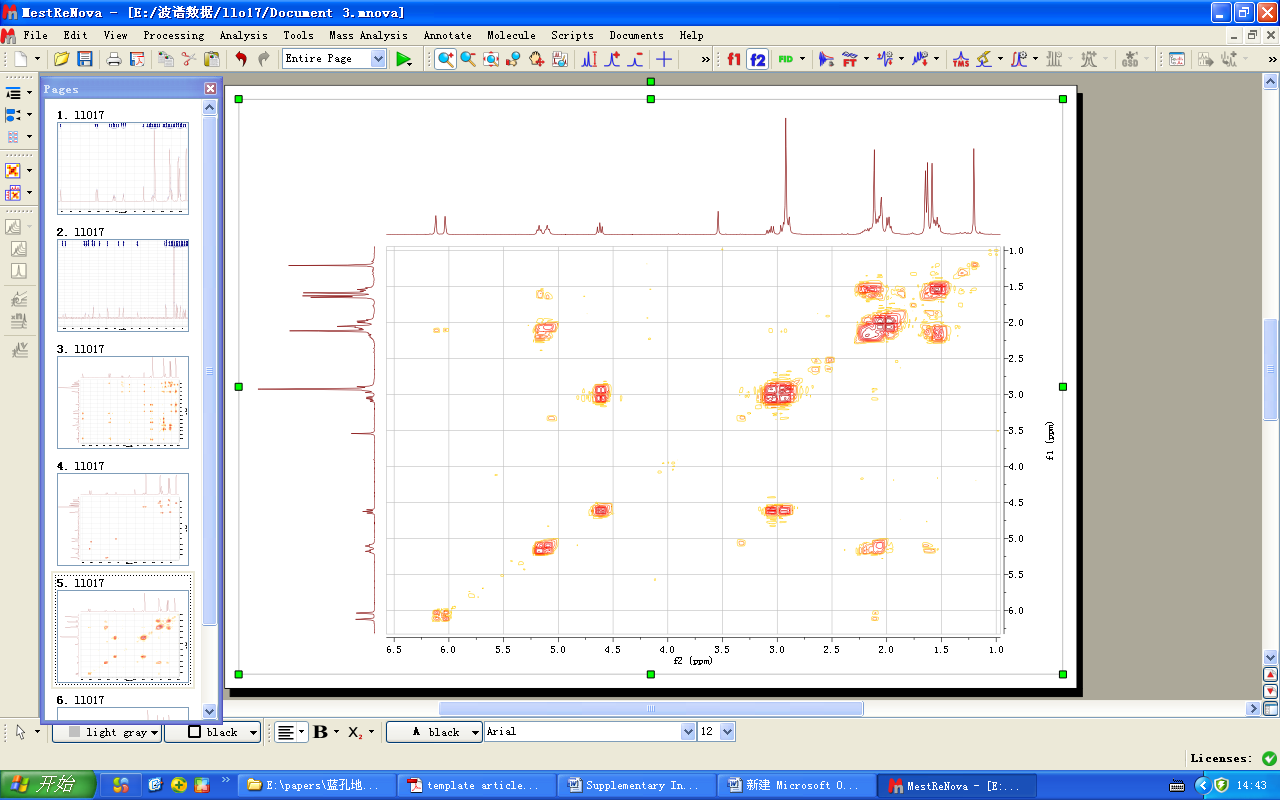


ROESY spectrum for (9*S*,10*R*)-6,9-epoxy-10-hydroxyneogrifolin (**5**)


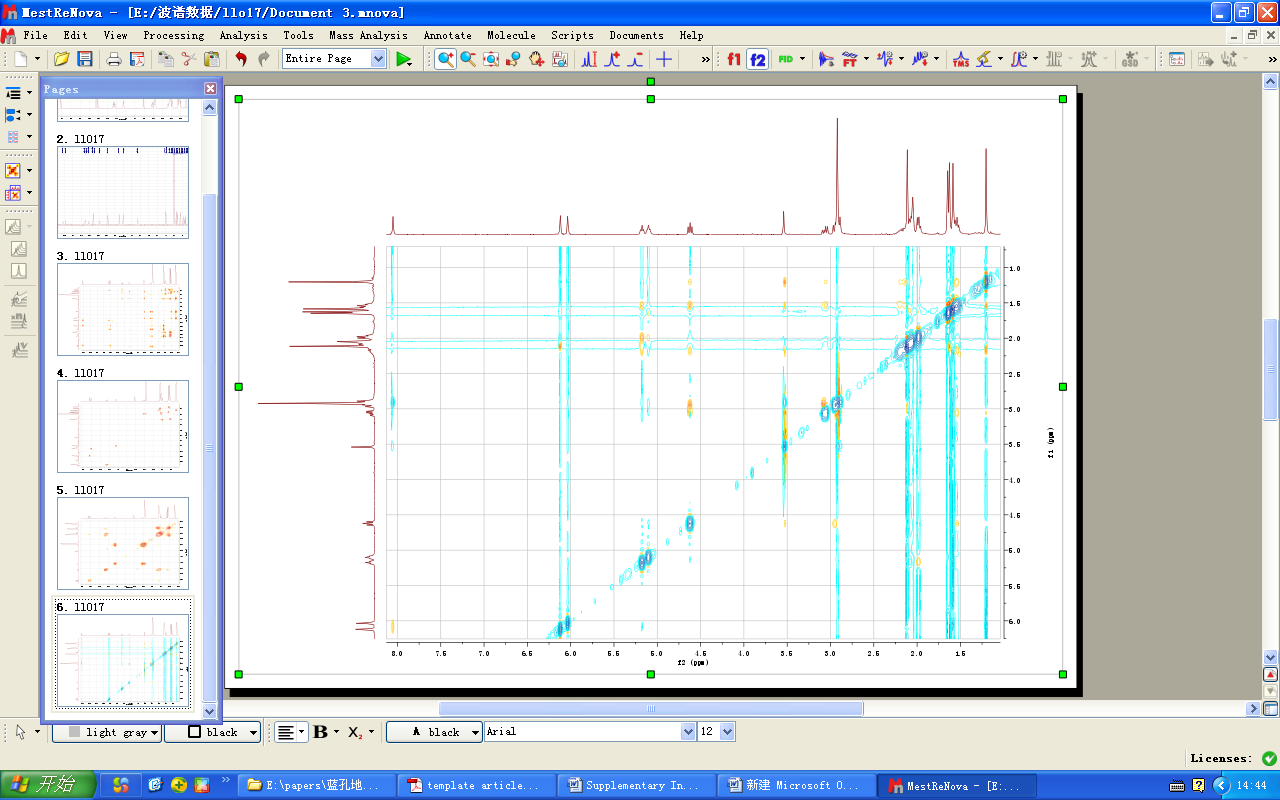


^1^H NMR spectrum for (−)-13,14-dihydroxyneogrifolin (**6**)


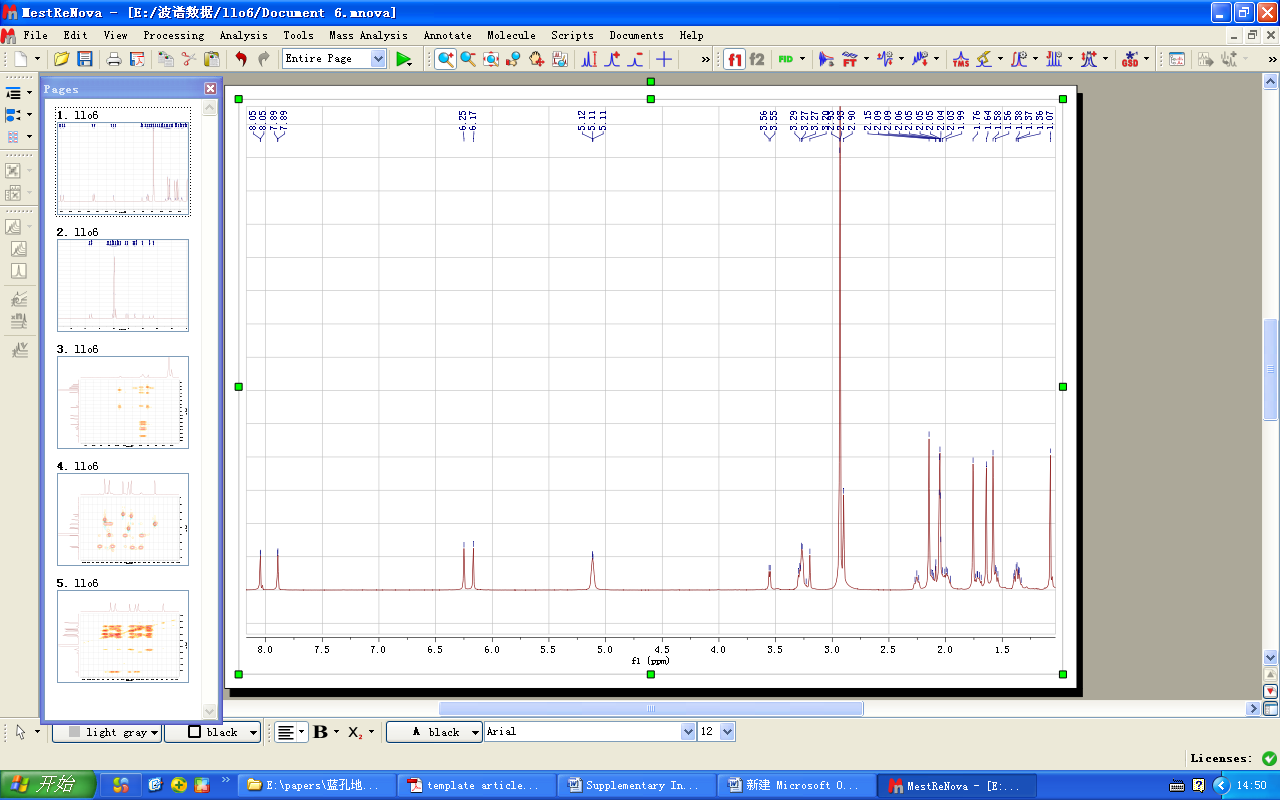


^13^C NMR spectrum for (−)-13,14-dihydroxyneogrifolin (**6**)


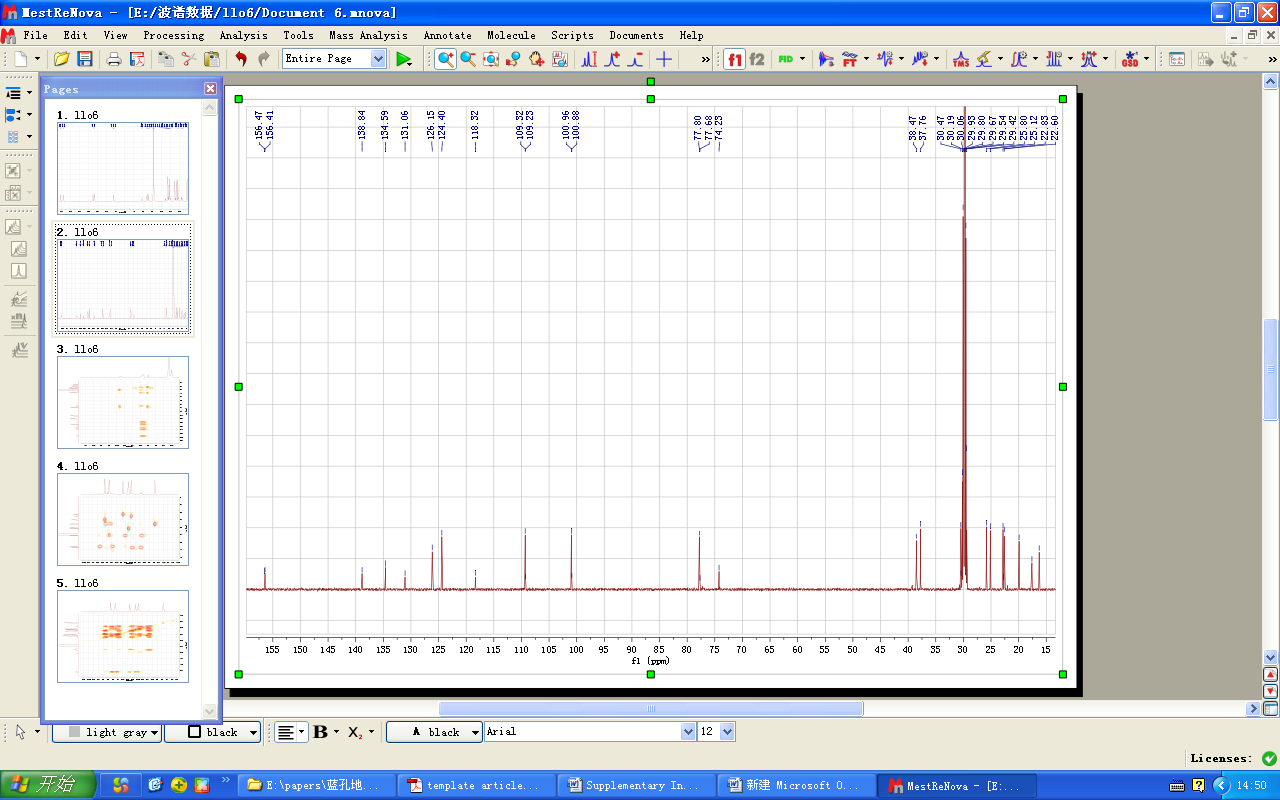


HSQC spectrum for (−)-13,14-dihydroxyneogrifolin (**6**)


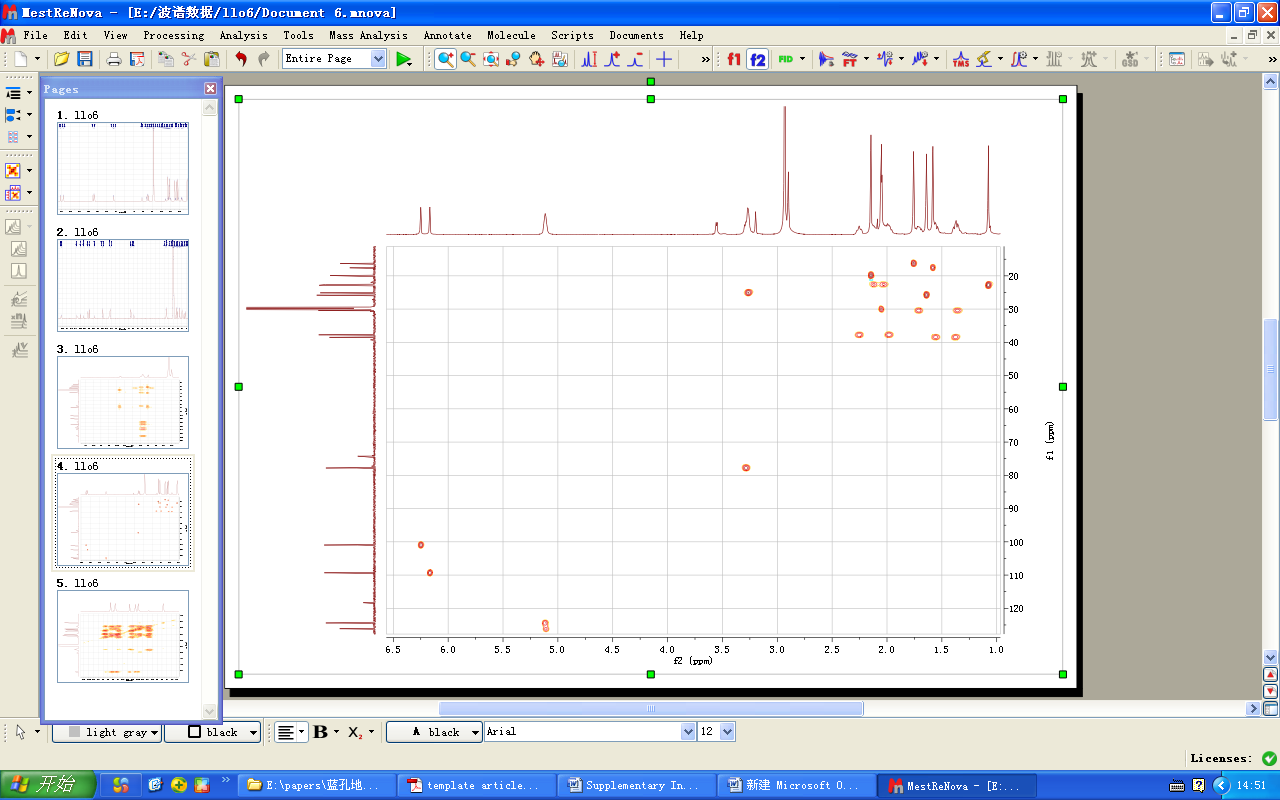


HMBC spectrum for (−)-13,14-dihydroxyneogrifolin (**6**)


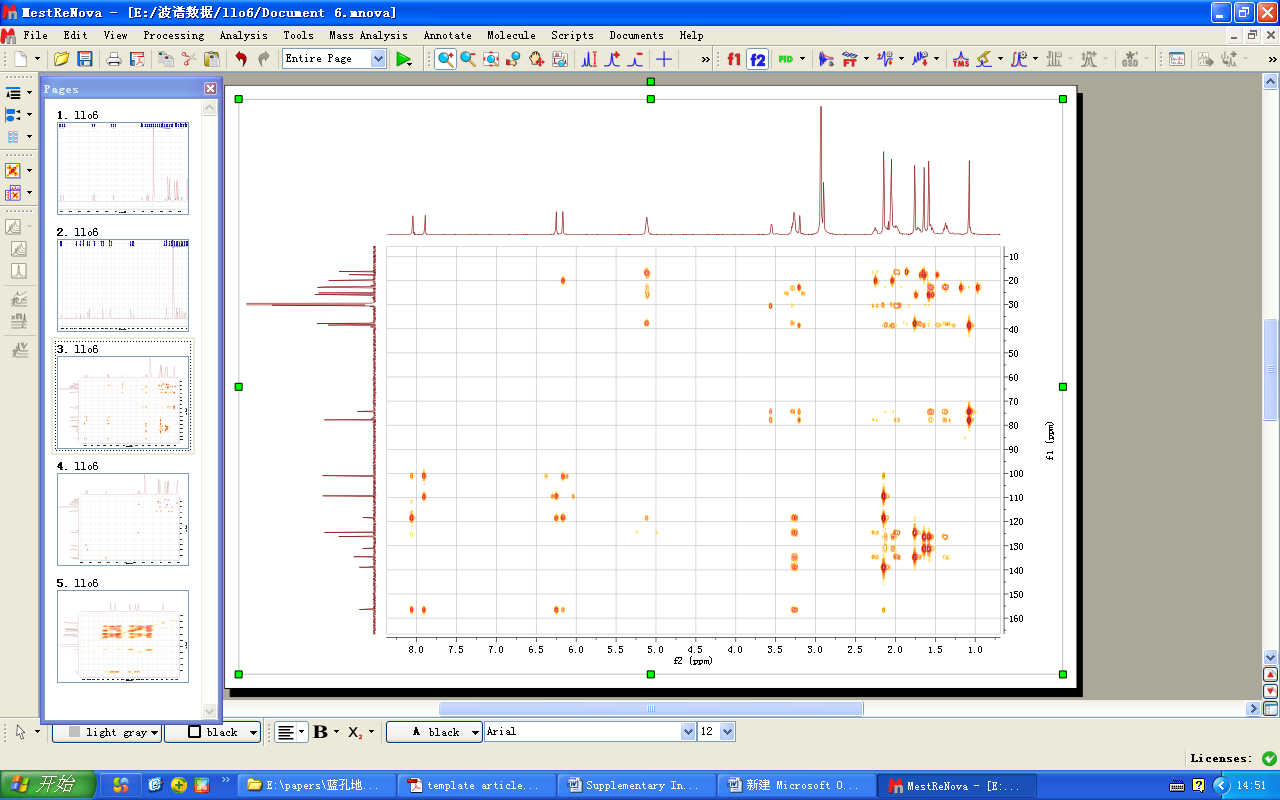


COSY spectrum for (−)-13,14-dihydroxyneogrifolin (**6**)


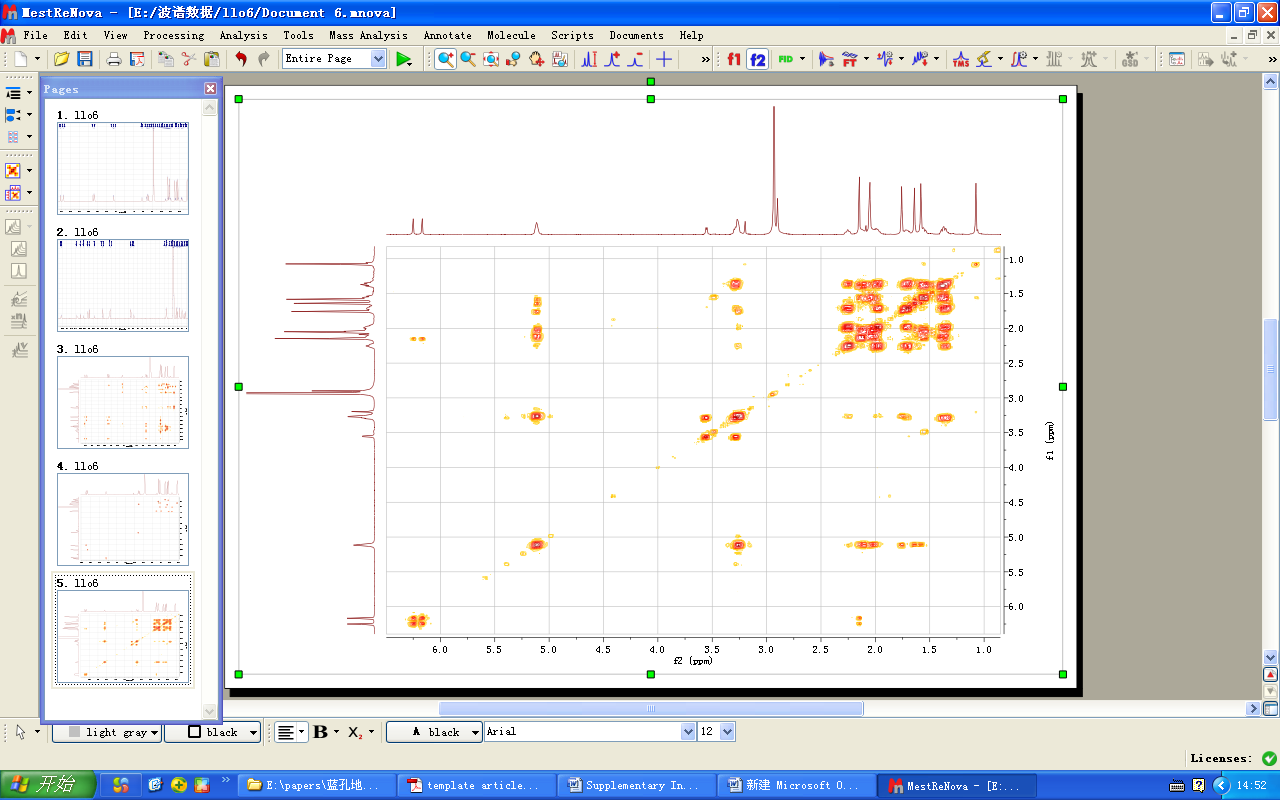


^1^H NMR spectrum for albatrelin G (**7**)


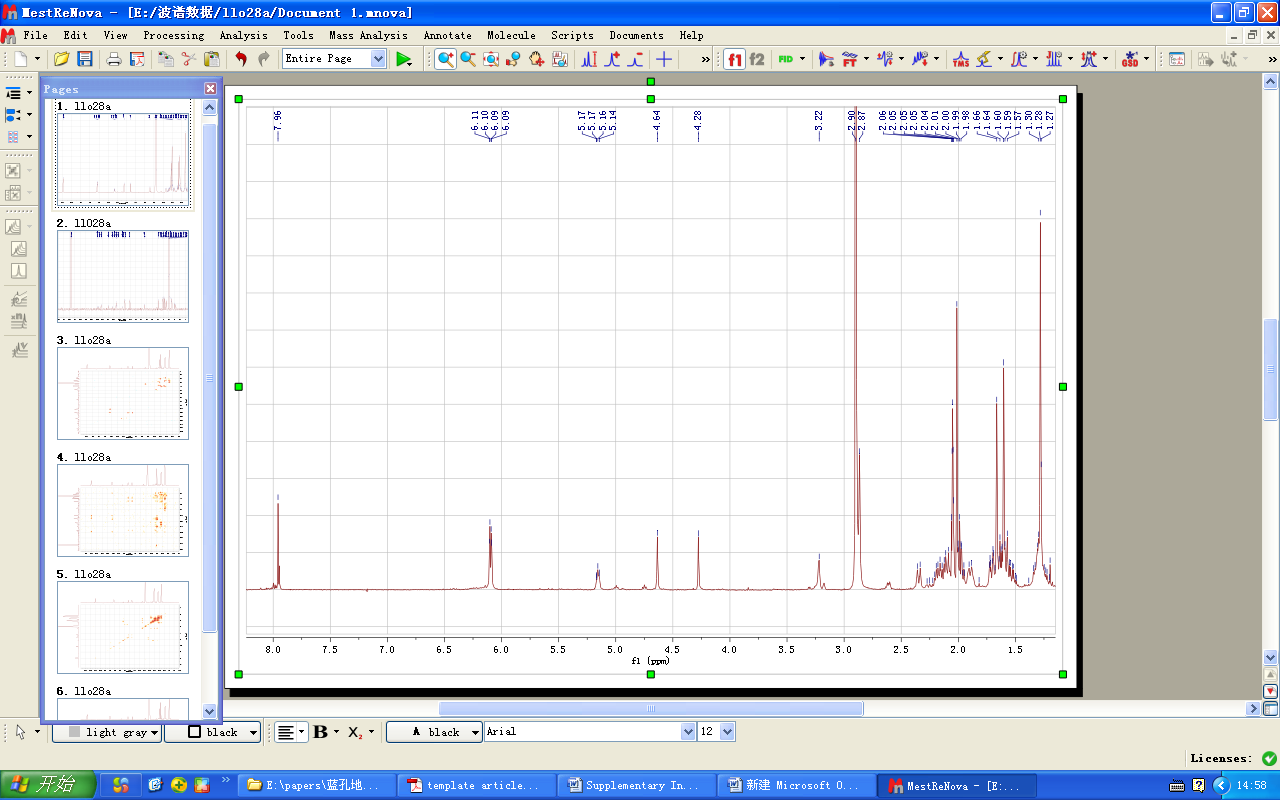


^13^C NMR spectrum for albatrelin G (**7**)


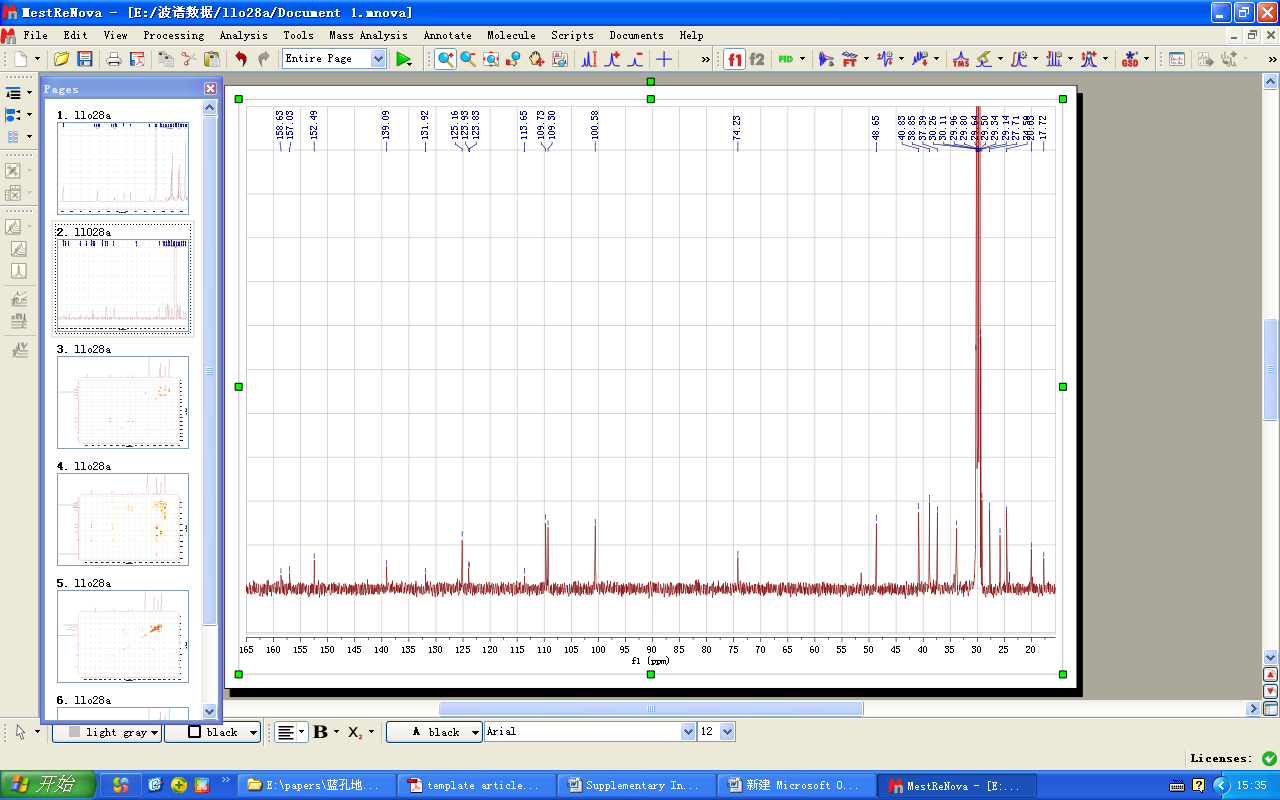


HSQC spectrum for albatrelin G (**7**)


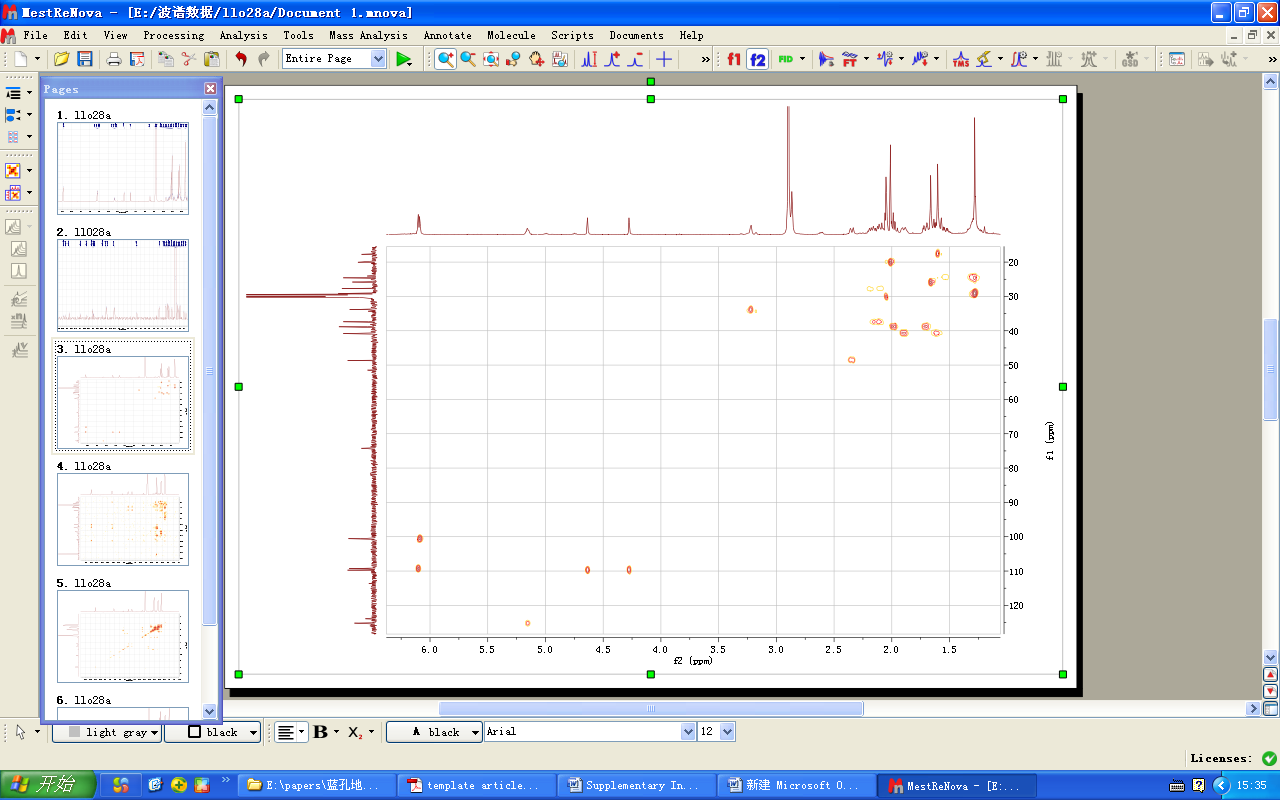


HMBC spectrum for albatrelin G (**7**)


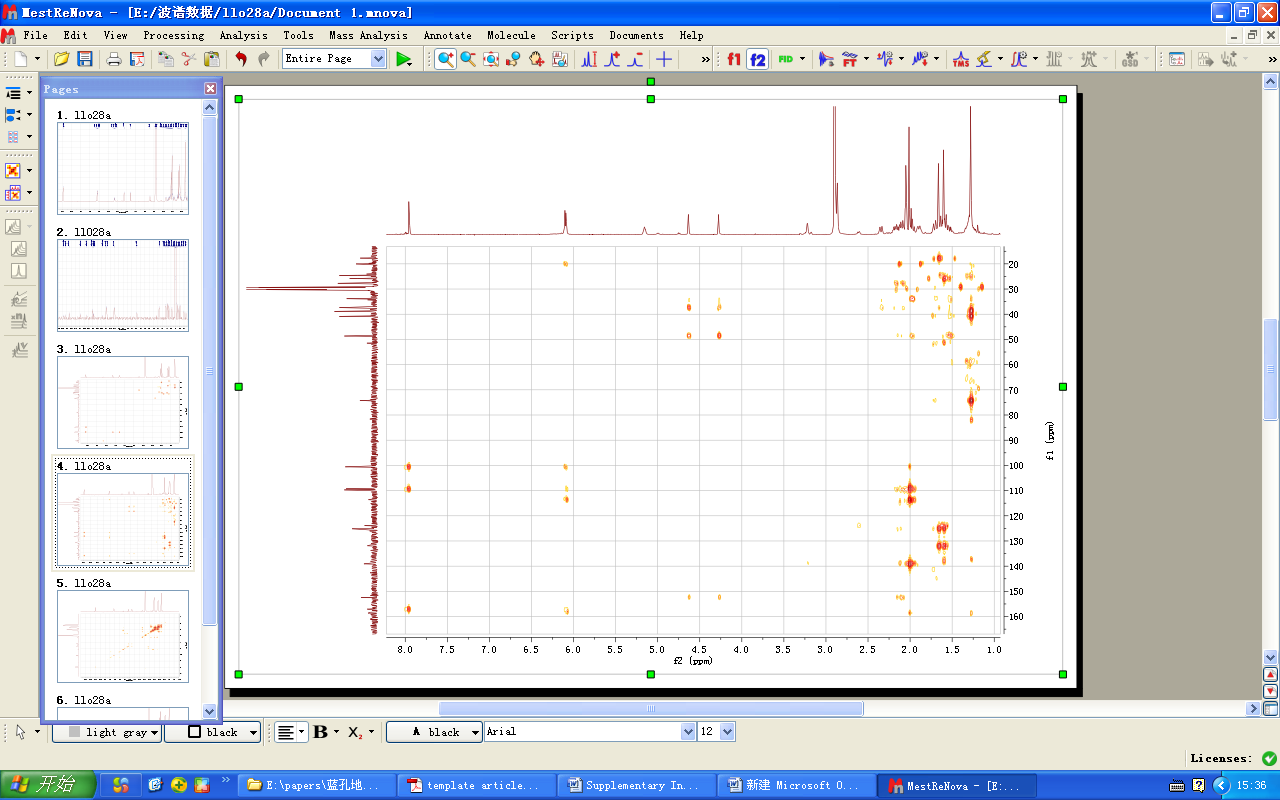


COSY spectrum for albatrelin G (**7**)


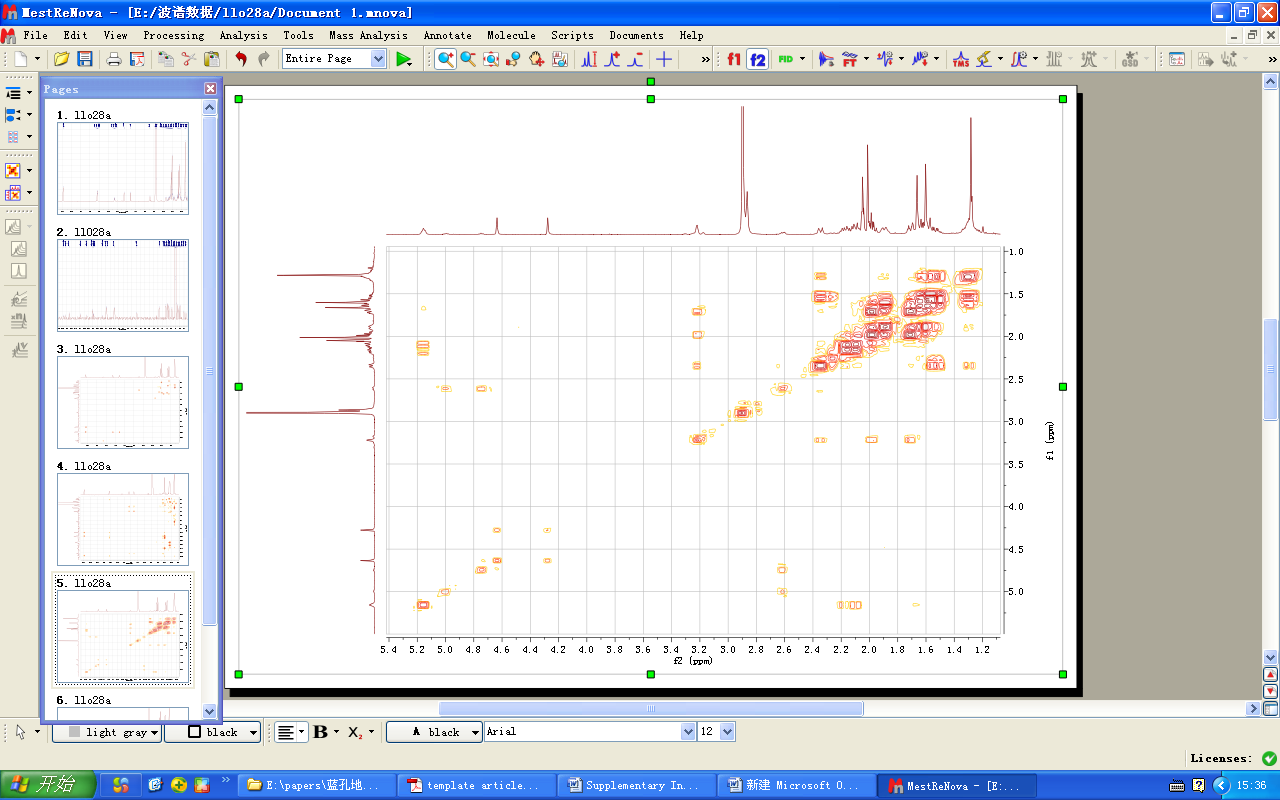


ROESY spectrum for albatrelin G (**7**)


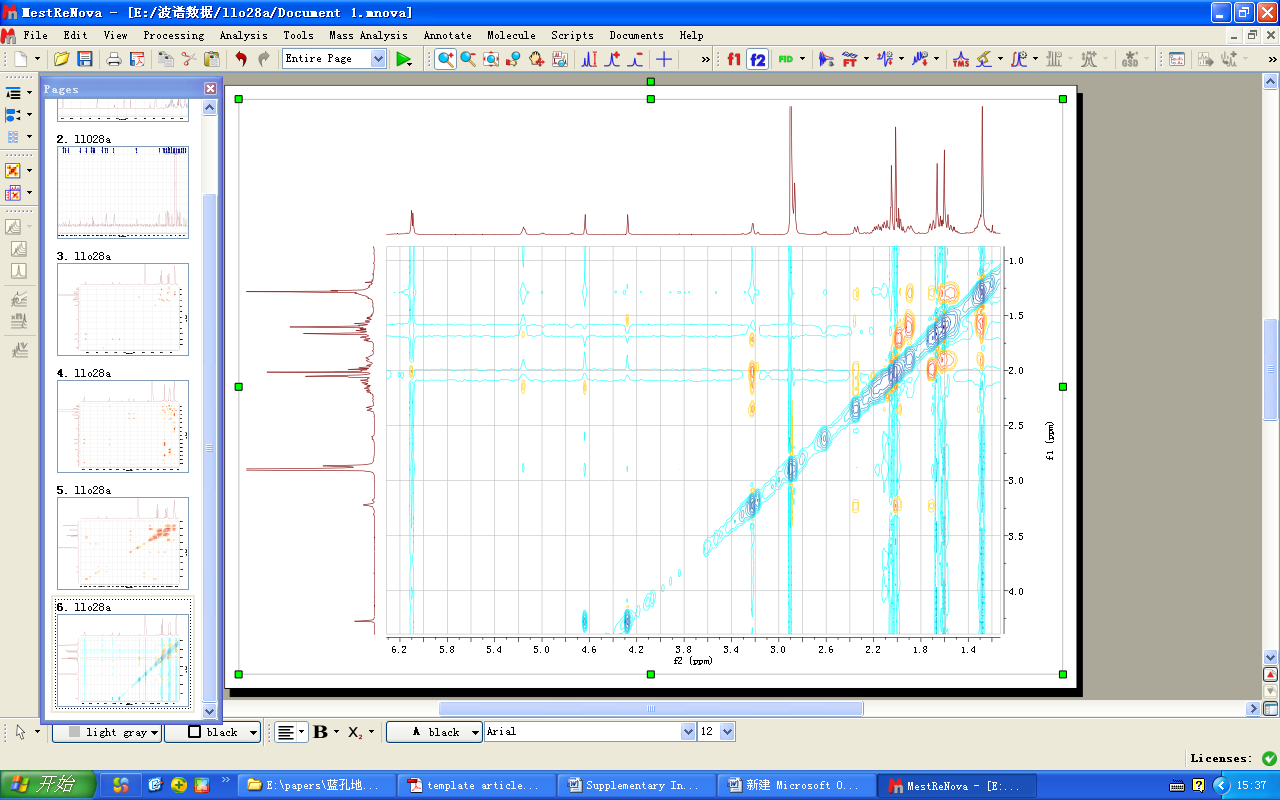


^1^H NMR spectrum for albatrelin H (**8**)


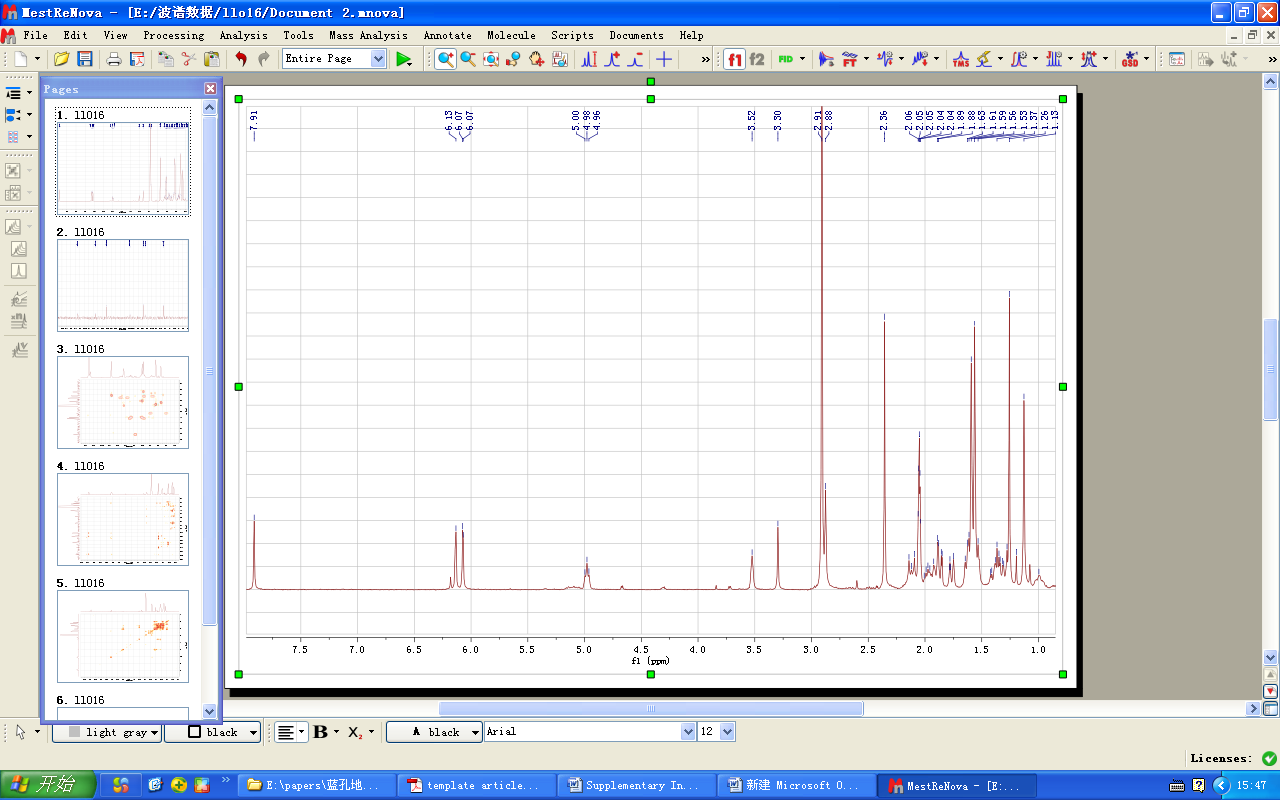


^13^C NMR spectrum for albatrelin H (**8**)


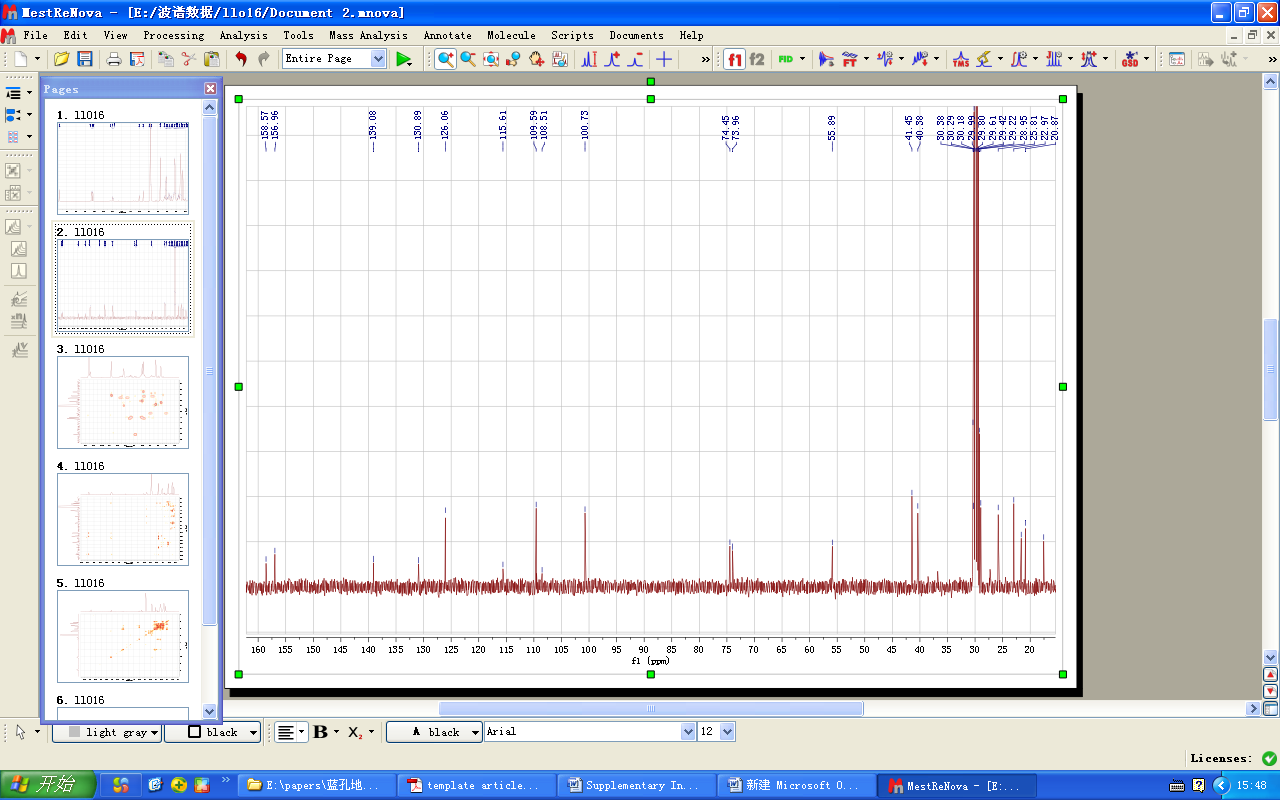


HSQC spectrum for albatrelin H (**8**)


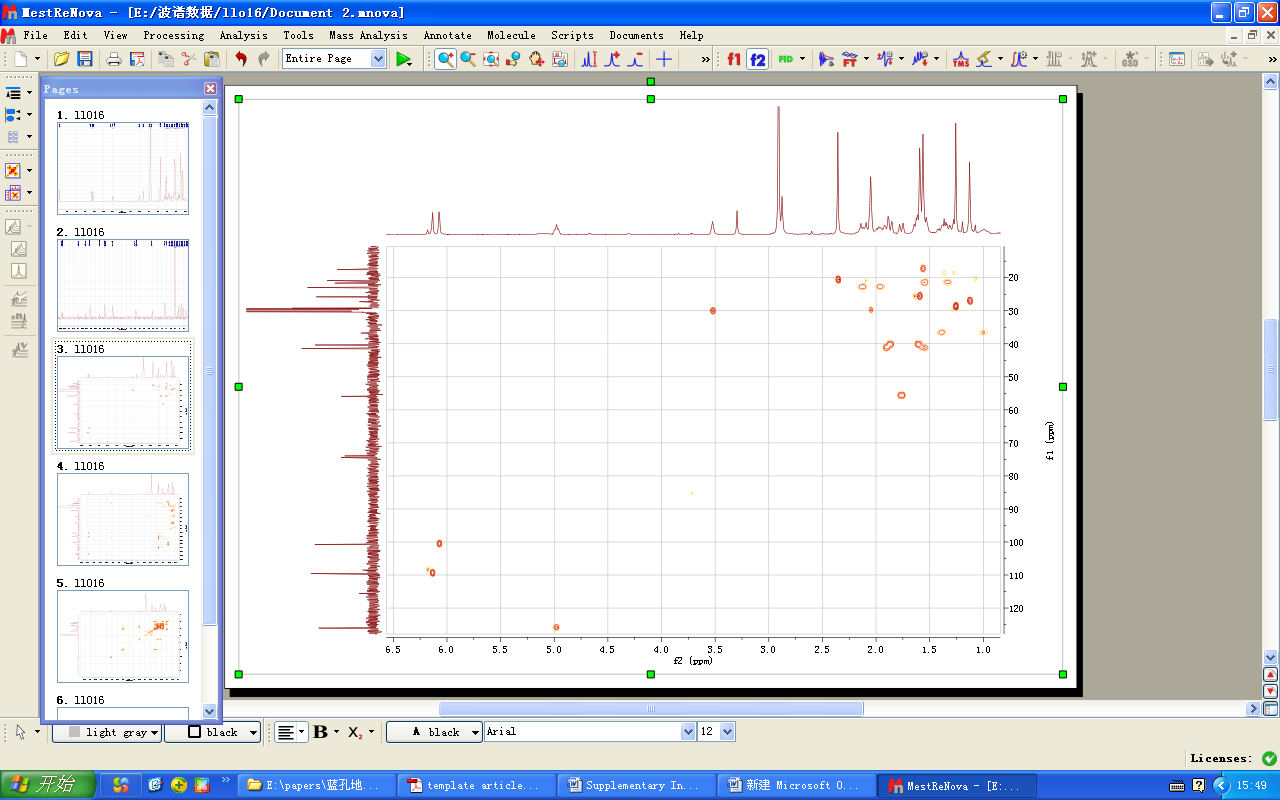


HMBC spectrum for albatrelin H (**8**)


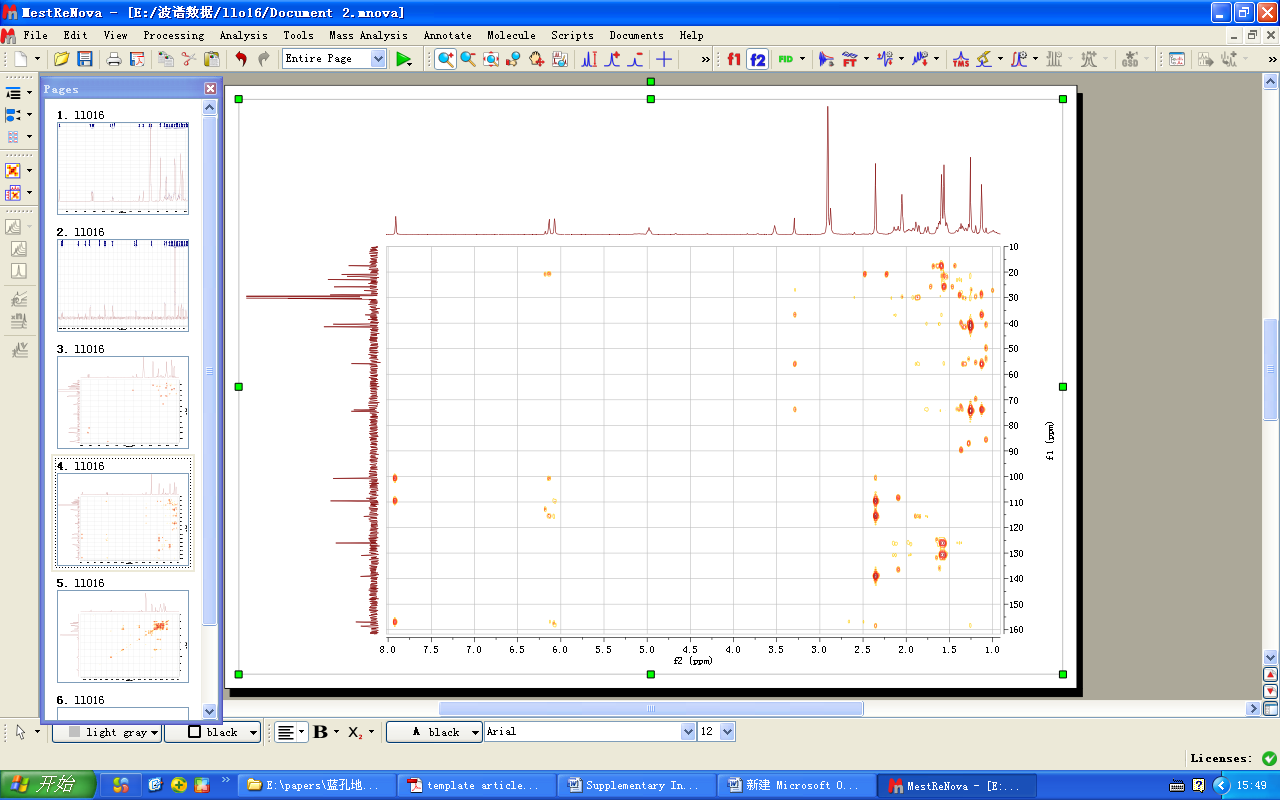


COSY spectrum for albatrelin H (**8**)


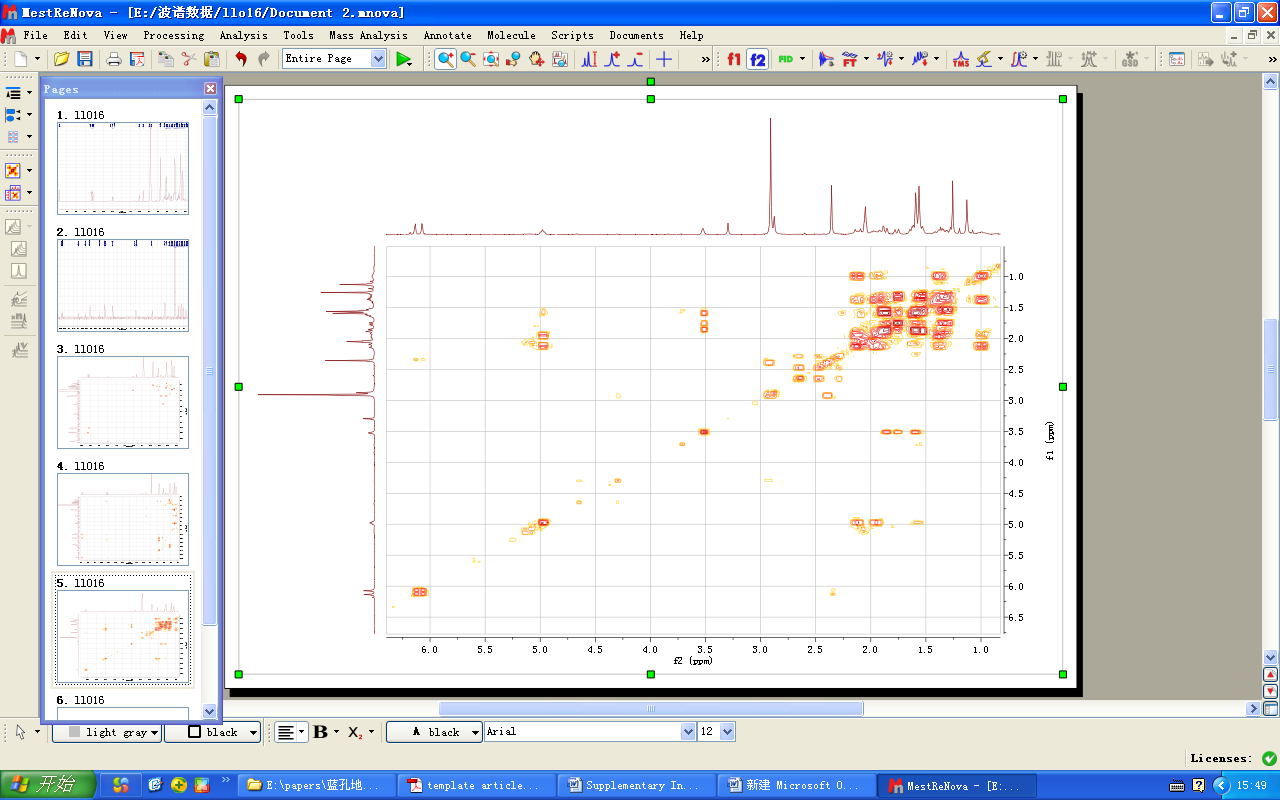


ROESY spectrum for albatrelin H (**8**)


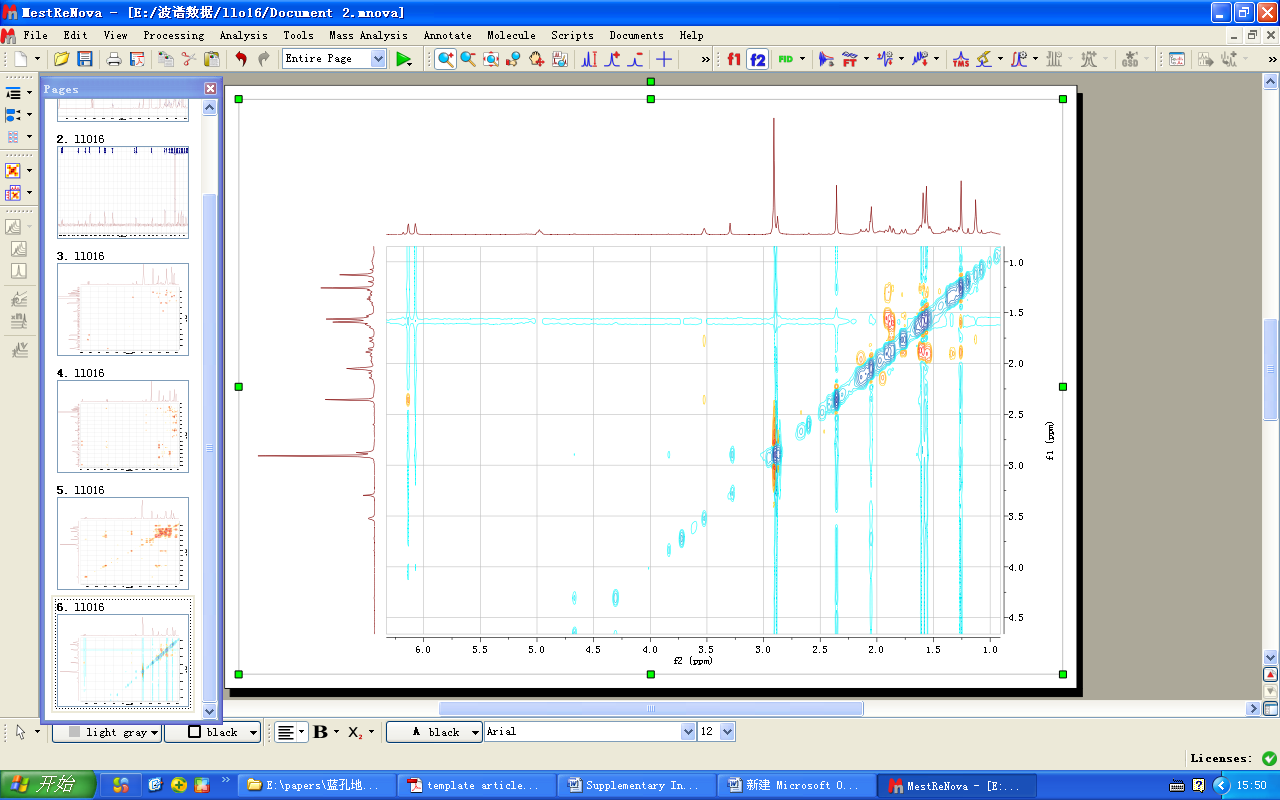


^1^H NMR spectrum for (*S*)-10-hydroxygrifolin (**9**)


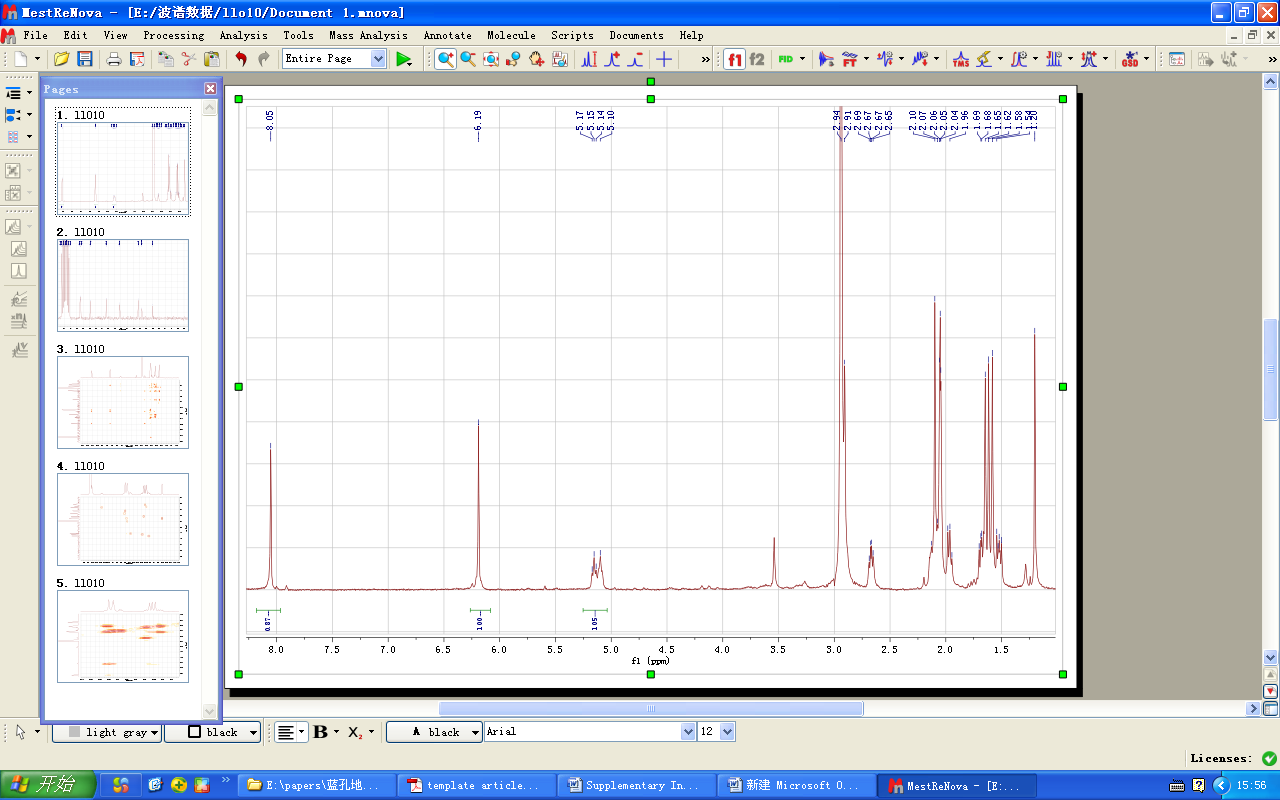


^13^C NMR spectrum for (*S*)-10-hydroxygrifolin (**9**)


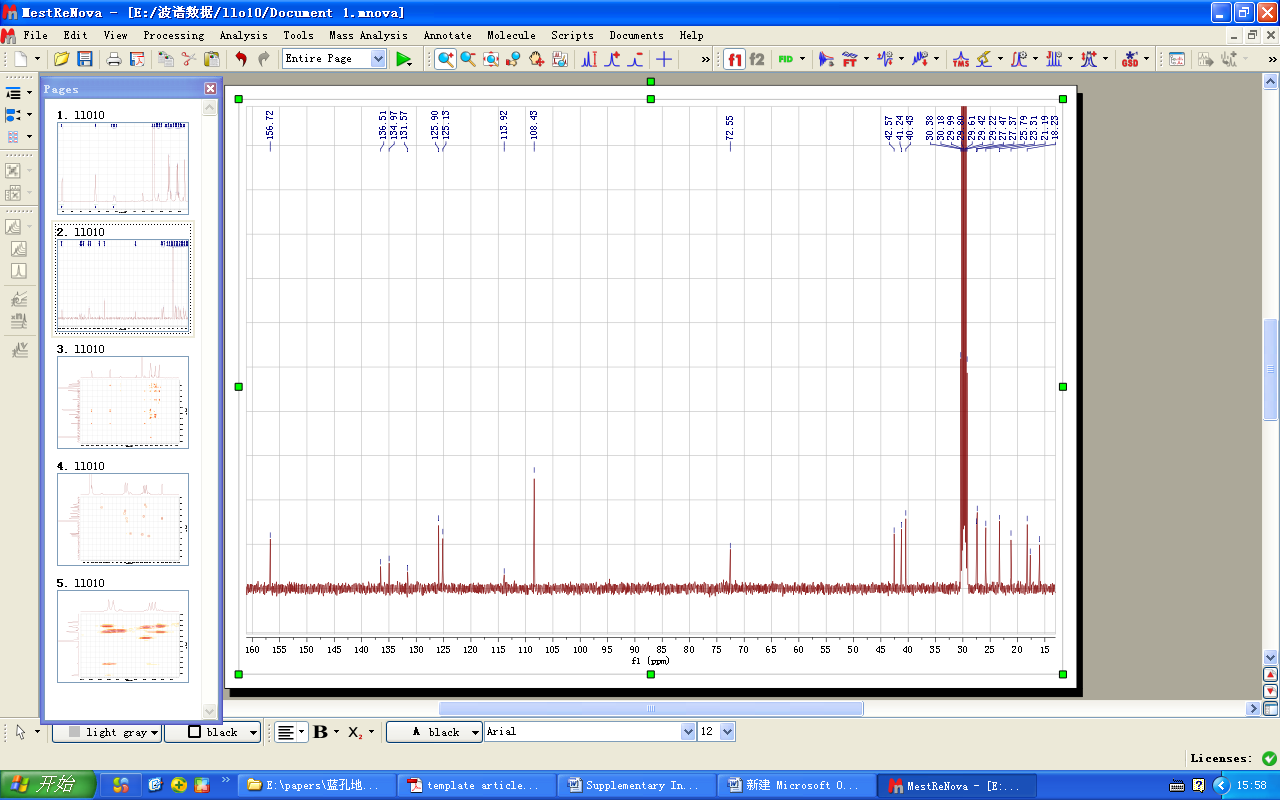


HSQC spectrum for (*S*)-10-hydroxygrifolin (**9**)


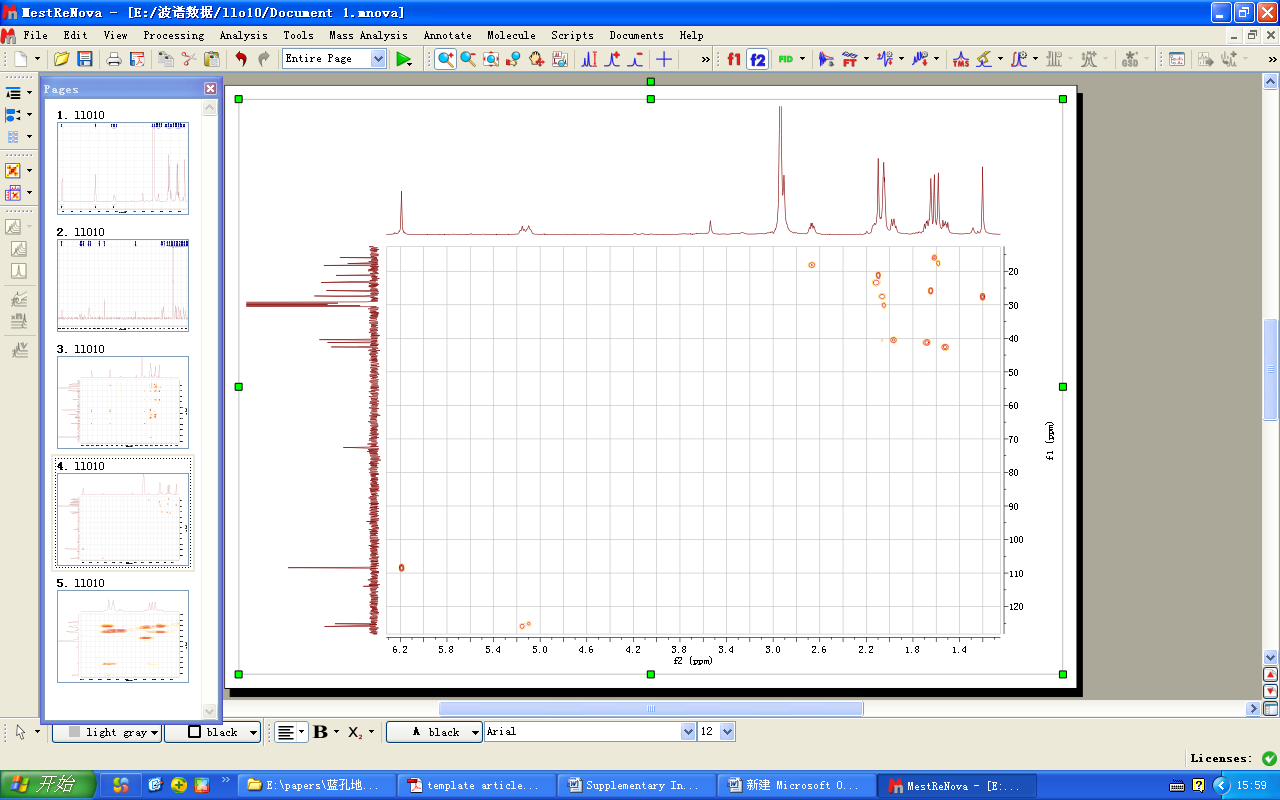


HMBC spectrum for (*S*)-10-hydroxygrifolin (**9**)


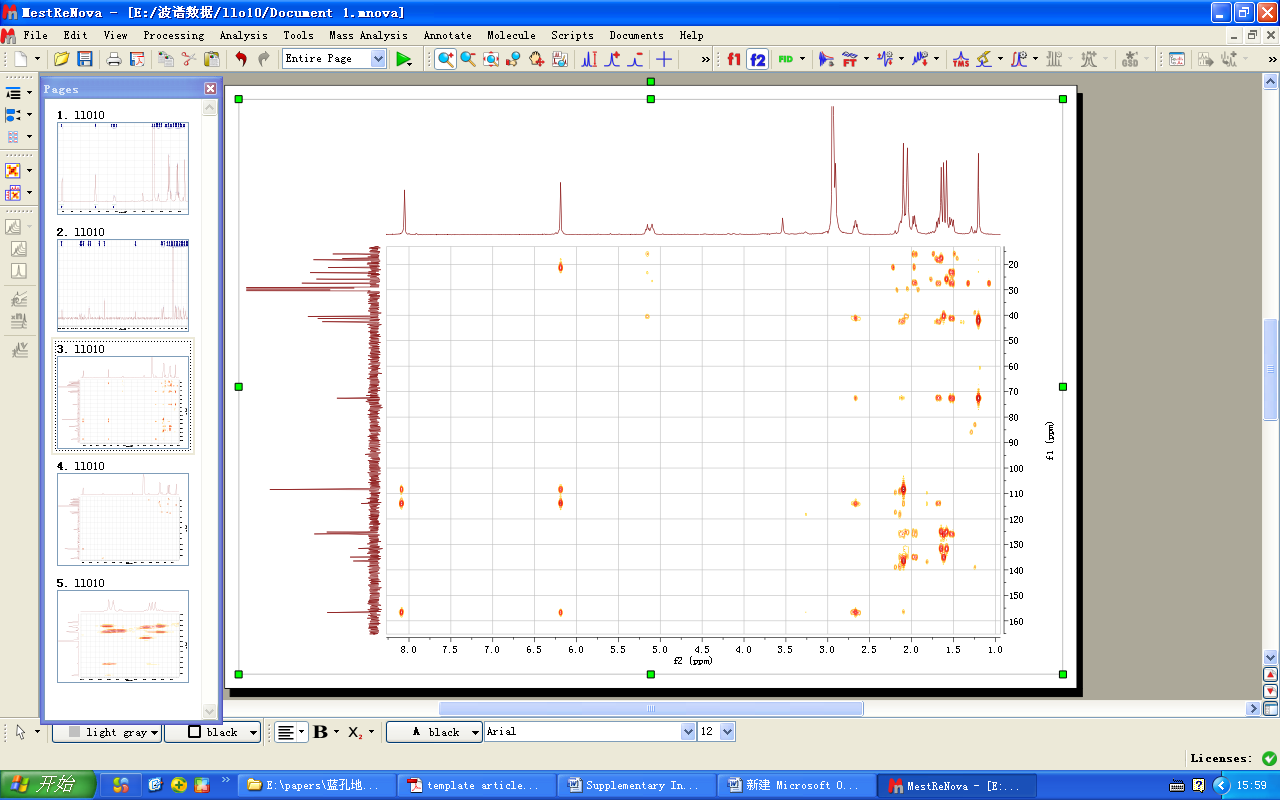


COSY spectrum for (*S*)-10-hydroxygrifolin (**9**)


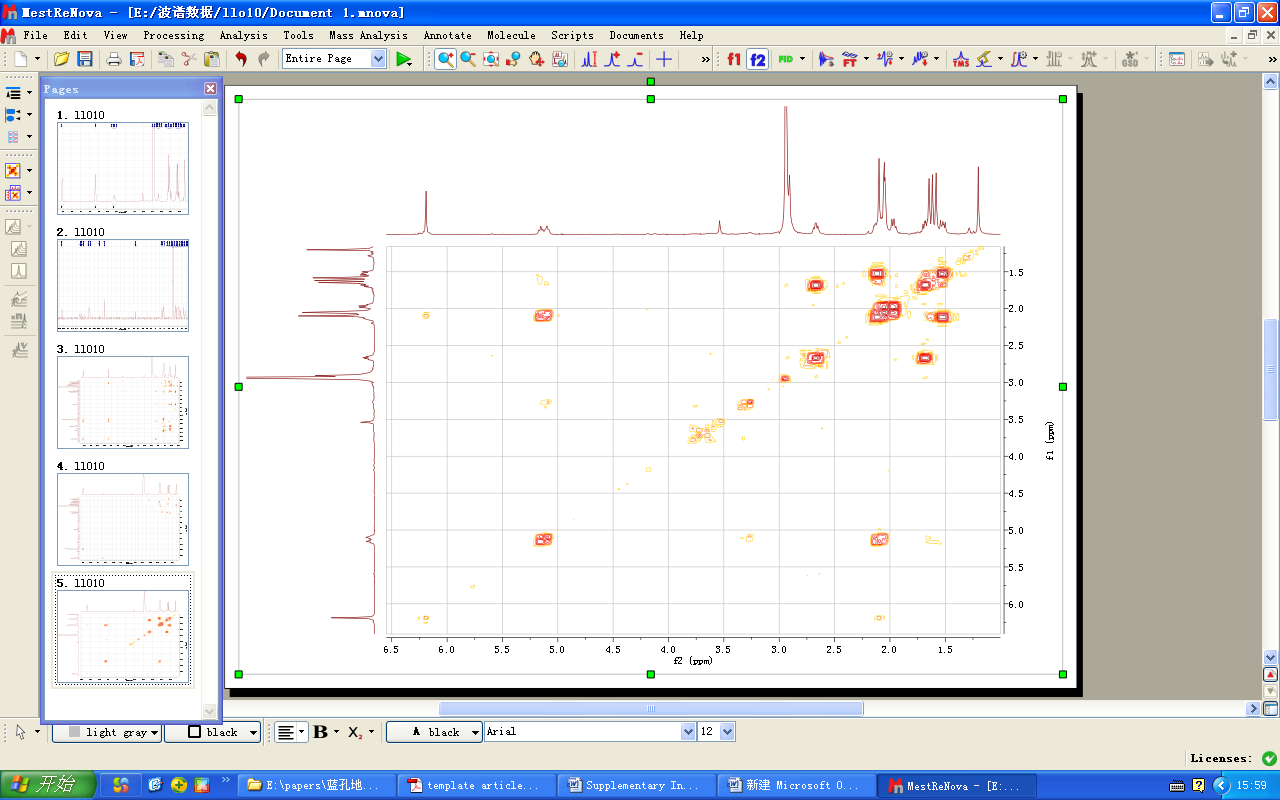


^1^H NMR spectrum for **6a**


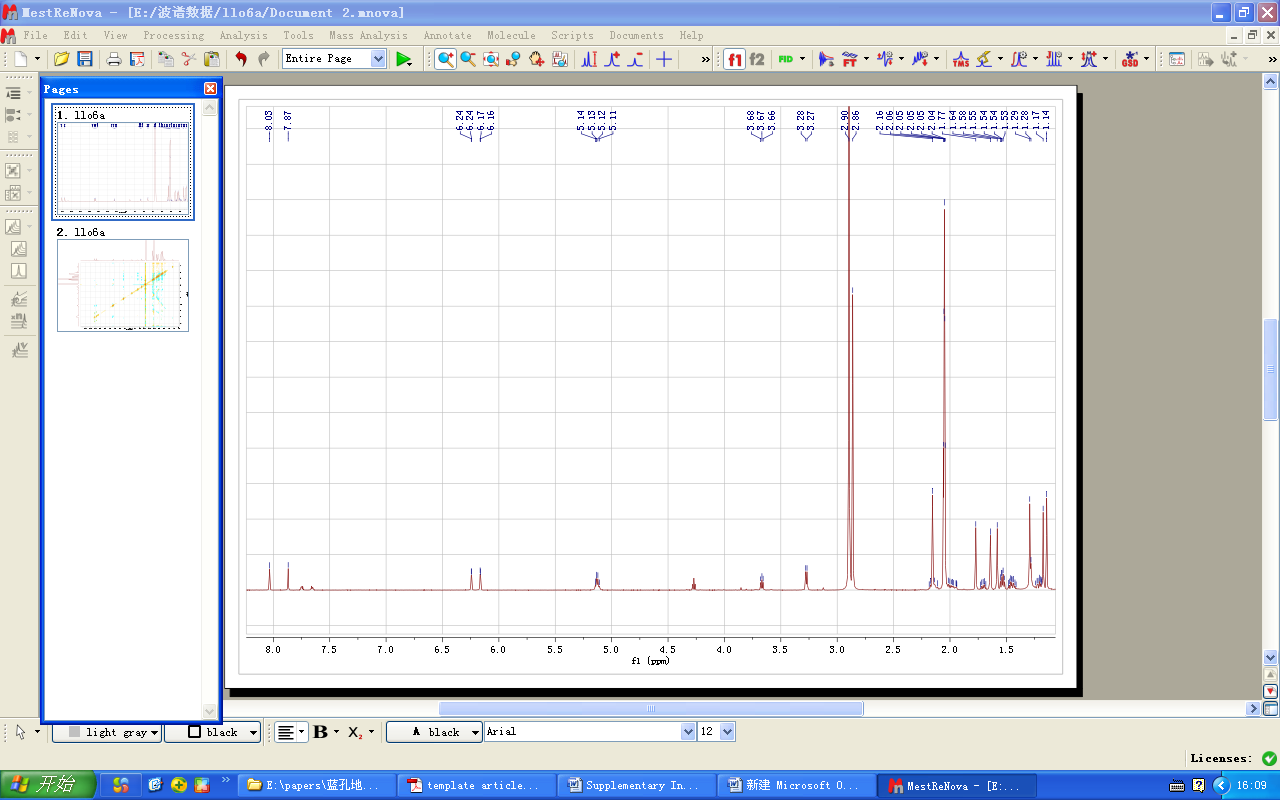


ROESY spectrum for **6a**


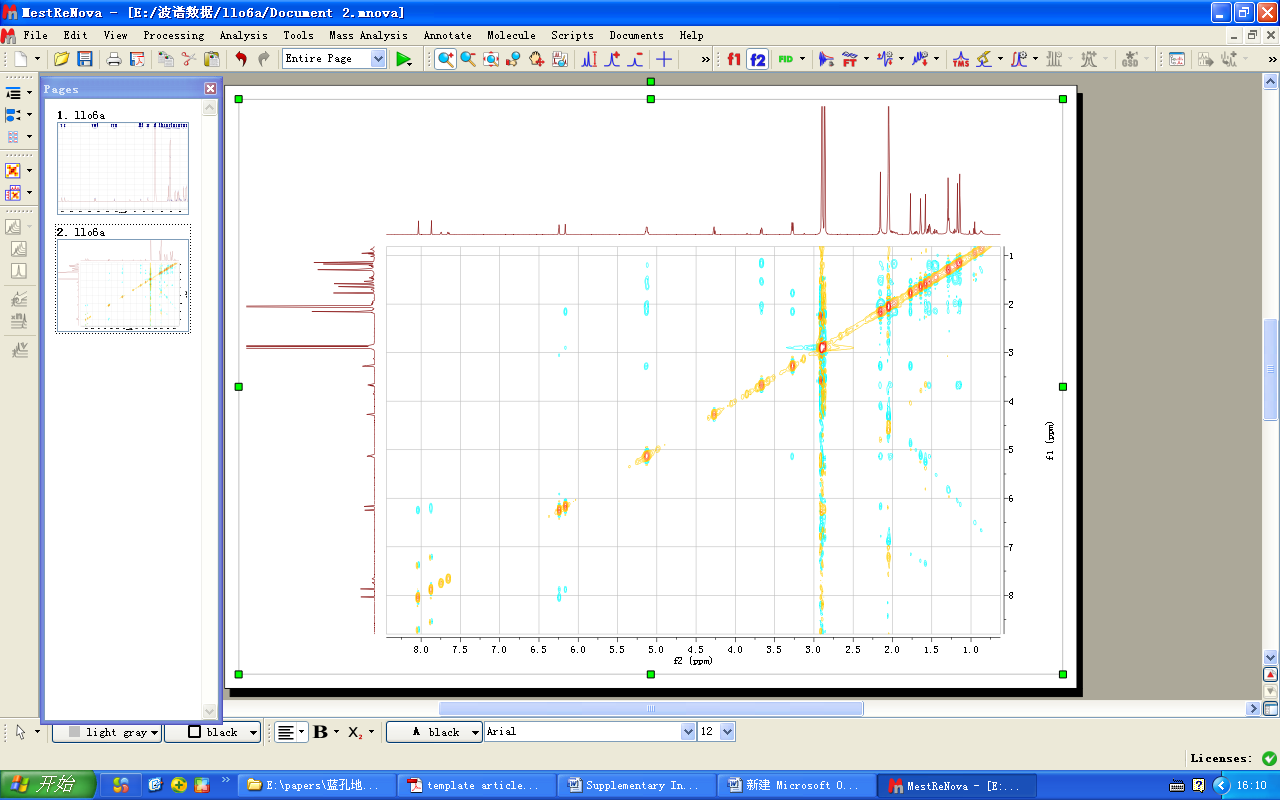

Supplement: Supplementary file 1 — Supplementary material 1 (DOC 8005 kb) [file 13659_2014_15_MOESM1_ESM.doc]
